# The Circadian–Melatonin Axis in Bone Remodeling: Receptor-Dependent Signaling, Receptor-Independent Actions, and Translational Constraints

**DOI:** 10.3390/biom16091232

**Published:** 2026-08-25

**Authors:** Ching-Chieh Lin, Yi-Chou Hou, Po-Jen Hsiao, Kuo-Cheng Lu

**Affiliations:** 1 Division of Pulmonary and Critical Care, Department of Internal Medicine, Taoyuan Armed Forces General Hospital, No. 168, Zhongxing Road, Longtan District, Taoyuan City 325, Taiwan; 2 School of Medicine, College of Medicine, Fu Jen Catholic University, New Taipei City 242062, Taiwan; 3 Division of Nephrology, Department of Internal Medicine, Cardinal Tien Hospital, School of Medicine, College of Medicine, Fu Jen Catholic University, New Taipei City 24205, Taiwan; 4 Division of Nephrology, Department of Internal Medicine, Tri-Service General Hospital, National Defense Medical Center, Taipei 114, Taiwan; 5 Department of Life Sciences, National Central University, Taoyuan 320, Taiwan; 6 Division of Nephrology, Department of Internal Medicine, Taoyuan Armed Forces General Hospital, Taoyuan 325, Taiwan; 7 Division of Nephrology, Department of Medicine, Taipei Tzu Chi Hospital, Buddhist Tzu Chi Medical Foundation, New Taipei City 23143, Taiwan; 8 Division of Nephrology, Department of Internal Medicine, Fu Jen Catholic University Hospital, New Taipei City 24352, Taiwan

**Keywords:** bone remodeling, chronotherapy, circadian rhythm, KEAP1–NRF2 signaling, melatonin, MT2 receptor, OPG/RANKL axis, osteoporosis, receptor-independent mechanisms, Wnt/β-catenin signaling

## Abstract

Bone remodeling is rhythmically regulated, yet the contribution of the circadian–melatonin axis to osteoporosis remains incompletely defined, in part because mechanistic findings obtained at high experimental concentrations are frequently extrapolated to physiological signaling. This narrative review examines that inference. PubMed/MEDLINE, Embase, Scopus, and Web of Science were searched from inception to July 2026 for English-language studies of melatonin, circadian clock genes, and bone; molecular, preclinical, epidemiological, and clinical evidence was appraised with attention to receptor dependence, exposure concentration, and study architecture. In osteoblast-lineage cells, melatonin promotes osteogenic differentiation through MT2-linked Wnt/β-catenin and MEK1/2–MEK5 signaling, post-translational stabilization of SP7, and modulation of the OPG/RANKL axis. By contrast, direct antiosteoclastic and antioxidant effects are usually reported at micromolar concentrations, four to six orders of magnitude above nocturnal plasma levels, and are increasingly attributable to receptor-independent chemistry converging on the ROS–KEAP1–NRF2 node shared with structurally unrelated antioxidant compounds. This exposure mismatch suggests that conventional oral doses engage receptor-mediated osteoblast pathways rather than reproduce high-dose antiresorptive effects; sustained exposure at or above 1 µM is not attainable by conventional oral administration, and the chronic safety of the doses that would be required has not been characterized. In humans, bone resorption has an intrinsic circadian rhythm, and night-shift work is associated with adverse skeletal outcomes, although causality remains unresolved. The five available randomized trials are small and heterogeneous; none was powered for fracture prevention, and none compared administration times for a skeletal endpoint. Melatonin therefore cannot currently be recommended for the treatment of osteoporosis. Human bone and marrow pharmacokinetics, receptor-specific in vivo dose–response experiments, and adequately powered monotherapy trials in established primary osteoporosis are the studies that would change this assessment.

## 1. Introduction

Osteoporosis is a systemic skeletal disorder characterized by reduced bone strength arising from low bone mass and microarchitectural deterioration, with a consequent increase in fragility-fracture risk. The Global Burden of Disease 2019 fracture analysis estimated 178 million new fractures worldwide in 2019 (95% uncertainty interval, 162–196 million) and 455 million prevalent cases of acute or long-term fracture symptoms [1]. Hip-fracture incidence is projected to increase substantially with population aging [2]. Existing pharmacotherapies reduce fracture risk, but suboptimal adherence, cost, contraindications, and rare serious adverse events leave an important treatment gap [3].

Conventional etiologic models emphasize sex-steroid deficiency, aging, and mechanical loading [4]. More recently, rotating shift work, transmeridian travel, and artificial light at night (ALAN) have been proposed as contributors to skeletal disease. The rationale comprises several links that differ substantially in evidentiary strength: human bone resorption is endogenously rhythmic; skeletal clocks regulate gene expression in rodent bone; melatonin is a major hormonal signal of biological night and is suppressed by nocturnal light; melatonin alters bone-cell behavior in vitro and in animals; and some cohorts associate night-shift work with osteoporosis or fracture. The final inference—that restoring melatonin signaling will preserve human bone—is the least established and should not be treated as a demonstrated therapeutic principle.

The review is organized around three unresolved questions. First, are the skeletal effects demonstrated in vitro receptor-mediated at concentrations attainable in humans, or do they reflect receptor-independent chemistry at exposures that oral dosing cannot reproduce? Second, can phenotypes generated in clock-gene models be attributed to melatonin at all, given that several widely used inbred strains lack a rhythmic pineal melatonin signal? Third, are the human rhythmicity data sufficient to support chronotherapeutic targeting, particularly for the sclerostin-based arguments that appear in the literature? Existing reviews generally aggregate melatonin’s skeletal effects without separating receptor-dependent from receptor-independent actions or reporting the exposure at which each effect was observed; both distinctions are applied systematically here. The review therefore (i) delineates the molecular architecture of the circadian–melatonin axis in bone, separating receptor-dependent from receptor-independent actions and genetic evidence from cell-line observations; (ii) examines the exposure mismatch that constrains mechanistic interpretation; (iii) appraises the human evidence with attention to sample size, intervention composition, and endpoint selection; and (iv) identifies the experiments required before chronobiological stratification could become clinically actionable.

### Evidence Identification and Appraisal

PubMed/MEDLINE, Embase, Scopus, and Web of Science were searched from inception to July 2026 using four Boolean concept blocks covering melatonin and kynuramine metabolites; circadian disruption and clock genes; bone-cell and skeletal outcomes; and shift work or light at night. Reference-list and forward-citation searches supplemented the database search. English-language publications were included. Eligibility was evidence-specific: mechanistic studies required reported melatonin concentrations or genetic models; clinical studies required randomized allocation and skeletal or musculoskeletal outcomes; and epidemiological studies required defined circadian-disruption exposures and skeletal outcomes. Conference abstracts, case reports, and studies lacking dose or concentration data were excluded from mechanistic claims. Screening was adjudicated by the authors, without formal risk-of-bias assessment or meta-analysis. Evidence classifications in Table 1, Table 2 and Table 3 describe study design and mechanistic strength and are not risk-of-bias measures.

## 2. Architecture of the Circadian–Melatonin Axis

### 2.1. Central Pacemaker and Peripheral Oscillators

**Figure 1:**
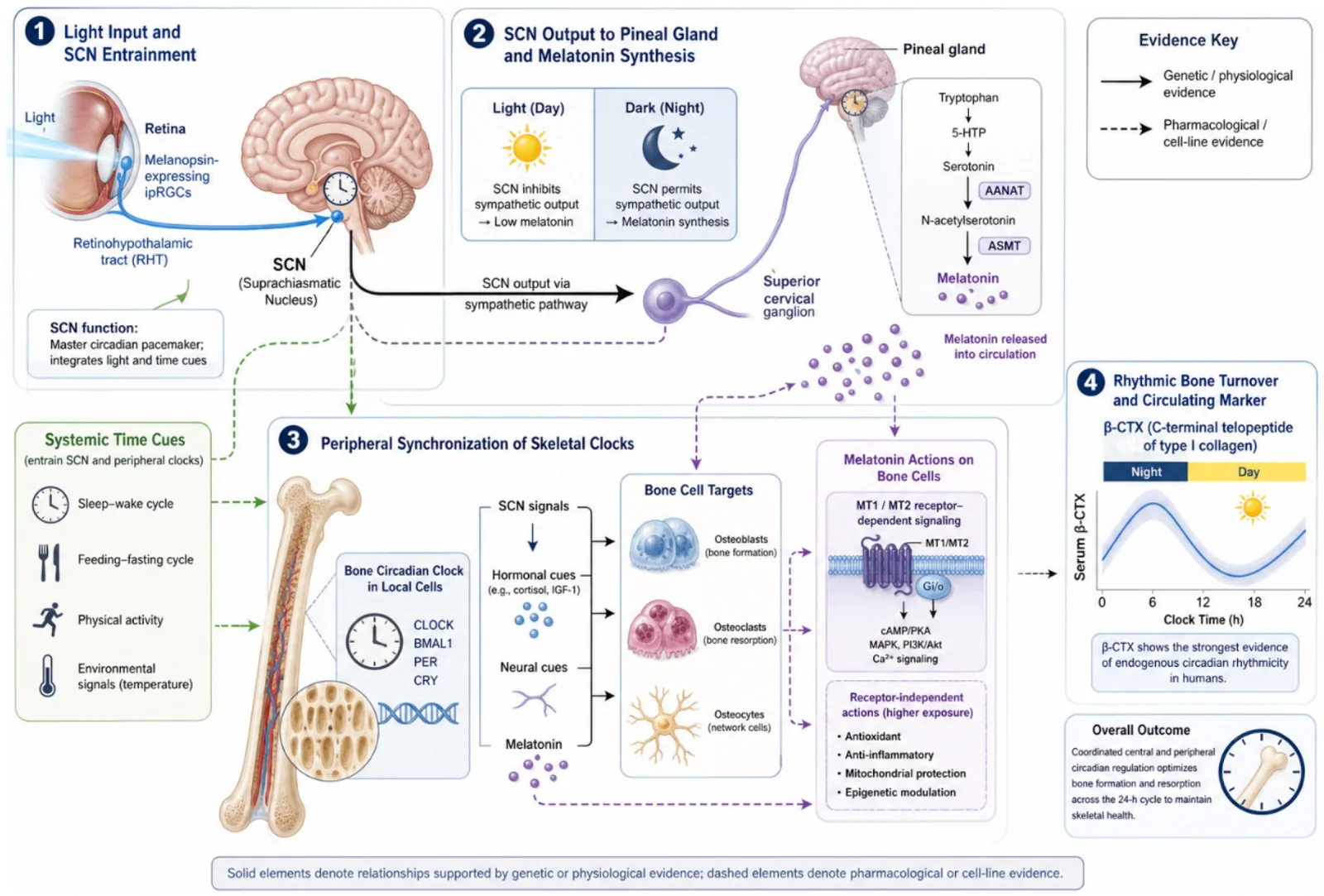
Central and peripheral organization of the circadian–melatonin axis in bone. Light detected by melanopsin-expressing ipRGCs entrains the SCN through the retinohypothalamic tract. In darkness, SCN-dependent sympathetic output permits pineal melatonin synthesis. The SCN and systemic time cues synchronize peripheral skeletal clocks, while melatonin may influence bone cells through MT1/MT2-dependent signaling and, at higher exposure, receptor-independent actions. Solid elements denote relationships supported by genetic or physiological evidence; dashed elements denote pharmacological or cell-line evidence. β-CTX is shown as the human bone-turnover marker with the strongest evidence of endogenous circadian rhythmicity.

The master circadian pacemaker resides in the suprachiasmatic nucleus (SCN) of the anterior hypothalamus. Photic input is conveyed primarily by melanopsin-expressing intrinsically photosensitive retinal ganglion cells (ipRGCs), which project through the retinohypothalamic tract [31]. In darkness, SCN output permits activation of a multisynaptic sympathetic pathway to the pineal gland through the superior cervical ganglion, enabling noradrenergic stimulation of melatonin synthesis. The SCN also entrains peripheral oscillators in most mammalian tissues, including bone [31] (Figure 1).

### 2.2. The Core Transcription–Translation Feedback Loop in Skeletal Cells (Table 1)

The positive limb of the mammalian clock comprises the basic helix–loop–helix PAS transcription factors BMAL1 and CLOCK, which heterodimerize and bind canonical E-box elements (5′-CACGTG-3′) in target promoters. Their targets include the negative regulators of the *PER* and *CRY* families. After PER and CRY proteins accumulate, they oligomerize, re-enter the nucleus, and repress CLOCK/BMAL1 activity, generating a self-sustained oscillation of approximately 24 h. An accessory loop, in which REV-ERBα/β and RORα/β/γ compete at ROR response elements, regulates transcription of the gene encoding BMAL1 and enhances robustness. Post-translational modifications—including CK1δ/ε- and CK2-mediated phosphorylation, acetylation, ubiquitination by β-TrCP and FBXL3, and O-GlcNAcylation—help determine circadian phase and amplitude. Species-specific gene symbols are used throughout according to the convention stated in the note to Table 1 [5].

Circadian timing also regulates cellular antioxidant capacity. Transcription of the gene encoding NRF2, the master regulator of the antioxidant response element (ARE), is driven by CLOCK/BMAL1 through an E-box conserved in the mouse, rat, and human proximal promoters; NRF2 protein and its occupancy at AREs are rhythmic, and this rhythm is lost in clock-mutant animals [6]. NRF2 in turn feeds back on the core loop, binding enhancer regions of *Cry2*, increasing *Cry2* expression, and repressing CLOCK/BMAL1-regulated E-box transcription, so that redox state and timekeeping are interlocked rather than merely parallel [7]. Antioxidant capacity in a peripheral tissue is therefore expected to be a clock output, providing a route by which circadian disruption could compromise skeletal redox defense independently of melatonin.

Genetic deletion studies establish that this machinery is functionally coupled to skeletal cell fate, but the phenotypes are strongly lineage dependent. Global Bmal1 deletion results in low bone mass and a reduced number of active osteoblasts and osteocytes [25]. By contrast, osteoclast-lineage Bmal1 deletion (Bmal1osc−/− generated with Ctsk-Cre) reduces Acp5 and Nfatc1 expression, impairs osteoclast differentiation, and produces a high-bone-mass phenotype [8]. Other clock genes were not materially altered in that model, suggesting that BMAL1 can regulate osteoclast activity partly independently of its role in maintaining molecular oscillation. Clock disruption in osteoblast-lineage cells also alters resorption, demonstrating bidirectional coupling between skeletal lineages [32]. Additional clock-mutant models show reduced bone density and increased apoptosis involving PDIA3 [9], whereas Per1/Per2-mutant mice exhibit high bone mass through loss of leptin-mediated sympathetic inhibition of osteoblasts [10].

These findings demonstrate that circadian-clock disruption does not shift bone mass in a uniform direction. The net skeletal phenotype depends on the targeted lineage, developmental timing, Cre driver, and experimental background. In particular, the high-bone-mass phenotype produced by osteoclast-lineage Bmal1 deletion should not be conflated with the low-bone-mass phenotype of global Bmal1 deficiency or with phenotypes arising from clock disruption in mesenchymal or osteoblast-lineage cells [8,33,34,35,36].

This distinction also constrains interpretation of rodent circadian literature. Many clock-gene studies were conducted in C57BL/6J or related backgrounds that lack an appreciable rhythmic pineal melatonin signal because of nonfunctional *AANAT and ASMT alleles* [37]. Phenotypes generated in these strains therefore cannot be attributed to endogenous rhythmic melatonin signaling unless melatonin competence is specifically demonstrated. Intrinsic clock control of bone and melatonin-mediated skeletal regulation are related but mechanistically distinct bodies of evidence and are considered separately throughout this review [37,38,39,40,41].

### 2.3. Melatonin Biosynthesis, Receptors and Downstream Coupling

Melatonin (N-acetyl-5-methoxytryptamine) is synthesized from tryptophan through serotonin, with AANAT catalyzing the formation of N-acetylserotonin and ASMT—formerly termed HIOMT—catalyzing the final methylation step. Circulating melatonin rises after dim-light melatonin onset and peaks during the biological night; a peak around 02:00–04:00 is common under conventional sleep schedules but varies with chronotype, sleep timing, age, and assay. Nocturnal light acutely suppresses pineal melatonin secretion through melanopsin-containing ipRGC signaling to the SCN and downstream autonomic projections to the pineal gland [42,43,44,45].

Canonical melatonin signaling is mediated by two high-affinity G-protein-coupled receptors: MT1 (MTNR1A) and MT2 (MTNR1B). Both receptors have been detected in osteoblasts and mesenchymal progenitors, whereas their expression in osteoclast precursors declines during RANKL-driven differentiation [24]. MT1 and MT2 commonly couple to Gαi and inhibit adenylyl cyclase; MT2 can additionally engage Gαq/phospholipase C and MAPK signaling.

The strongest genetic evidence for receptor specificity in bone comes from Sharan and colleagues [11]. Pineal-specific Tph1 deletion reduced circulating melatonin and produced low bone mass through an isolated reduction in bone formation, without a detectable change in resorption. Mtnr1b-null mice likewise had low bone mass because of reduced osteoblast number, whereas Mtnr1a-null mice had normal bone mass. Osteoblasts derived from Mtnr1b-null mice showed cell-intrinsic defects in proliferation, differentiation, and mineralization and failed to respond to added melatonin, providing direct evidence that the anabolic effect requires MT2 in that model. The same study reported that daily oral melatonin increased bone accrual during growth and attenuated gonadectomy-induced structural and biomechanical deterioration.

Receptor-dependent coupling has also been examined in primary human cells. In human mesenchymal stromal cell (MSC)/peripheral-blood-monocyte cocultures, melatonin promoted osteoblast differentiation and mineralization and inhibited osteoclastogenesis in layered, but not transwell, configurations. These effects were attenuated by the MT2-selective antagonist 4-P-PDOT and by MEK1/2 (PD98059) and MEK5 (BIX02189) inhibitors, implicating MT2-linked MEK signaling [13]. Melatonin also increased RUNX2 and decreased PPARγ in both configurations and increased the OPG/RANKL ratio mainly by reducing RANKL in layered cocultures.

Melatonin and its kynuramine metabolites N^1^-acetyl-N^2^-formyl-5-methoxykynuramine (AFMK) and N^1^-acetyl-5-methoxykynuramine (AMK) can directly scavenge reactive oxygen and nitrogen species, accumulate in mitochondria, and induce endogenous antioxidant enzymes, including superoxide dismutase and glutathione peroxidase [46]. These effects can occur independently of cell-surface receptors and are likely to contribute increasingly as experimental concentrations enter the micromolar range. The transcriptional program through which those enzymes are induced is set out in Section 3.6.

### 2.4. Exposure, Receptor Occupancy, and the Basis for Mechanistic Attribution

This subsection states the interpretive framework used throughout the review; later sections apply it by label and cross-reference rather than restating it. The classification assigned to each experimental system is given in the “Receptor dependence” and “Basis for attribution” columns of Table 2.

Two criteria are used. An effect is classified as receptor-dependent only where it is abolished or attenuated by receptor antagonism (for example 4-P-PDOT for MT2) [47], by receptor knockdown, or by genetic deletion of *Mtnr1a* or *Mtnr1b* [24]. Pathway inhibitors acting downstream of the receptor—noggin, PD98059, H89, Gö6976—define the pathway but not the initiating receptor, and studies using only such inhibitors are classified as unresolved [21]. An effect is classified as receptor-independent where receptor expression is absent or lost in the system studied, or where the exposure is so far above the range required for high-affinity binding that receptor mediation cannot account for it, and where a plausible receptor-independent mechanism is demonstrated.

Physiological nocturnal plasma melatonin concentrations in healthy young adults generally lie in the tens to low hundreds of picograms per milliliter; 60–150 pg/mL corresponds to approximately 0.26–0.65 nM. Because MT1 and MT2 are high-affinity receptors with binding affinities extending from the picomolar to low-nanomolar range, endogenous subnanomolar concentrations can produce biologically relevant receptor activation. However, receptor occupancy and apparent saturation depend on receptor subtype, receptor density, tissue context, signaling amplification, and assay design and therefore cannot be assigned a universal threshold. The four operational exposure categories used throughout this review are summarized (Table S1). These boundaries are operational rather than formally standardized and should be interpreted together with receptor occupancy, exposure duration, and assay context [48,49,50,51].

Many in vitro bone studies use 10 µM to 1 mM melatonin—approximately four to six orders of magnitude above physiological plasma concentrations and far above the range required for high-affinity receptor binding. Effects observed only at these concentrations should not be assigned to MT1/MT2 signaling without receptor antagonism, knockdown, or genetic deletion and may instead reflect direct radical scavenging or other receptor-independent chemistry. Accordingly, Table 2 reports the exposure used in each experimental system whenever it is relevant to mechanistic interpretation. These exposures are not attainable by conventional oral administration in humans; the pharmacokinetic argument and its safety implications are set out in Section 8.4.

Two observations may partially narrow, but do not eliminate, this exposure gap. Older rodent and ex vivo studies reported melatonin concentrations in bone marrow above those in peripheral blood and identified melatonin-synthetic capacity in marrow cells. However, robust human data have not established the magnitude or consistency of a bone-marrow-to-plasma concentration gradient. These observations therefore do not justify extrapolating skeletal effects observed at micromolar or millimolar concentrations to physiological endocrine exposure. Direct pharmacokinetic studies measuring melatonin simultaneously in plasma, bone tissue, and bone marrow after oral administration in humans represent a high-priority translational experiment [40,41,52].

### 2.5. Diurnal Rhythms of Bone Turnover: What Is and Is Not Established 

**Figure 2:**
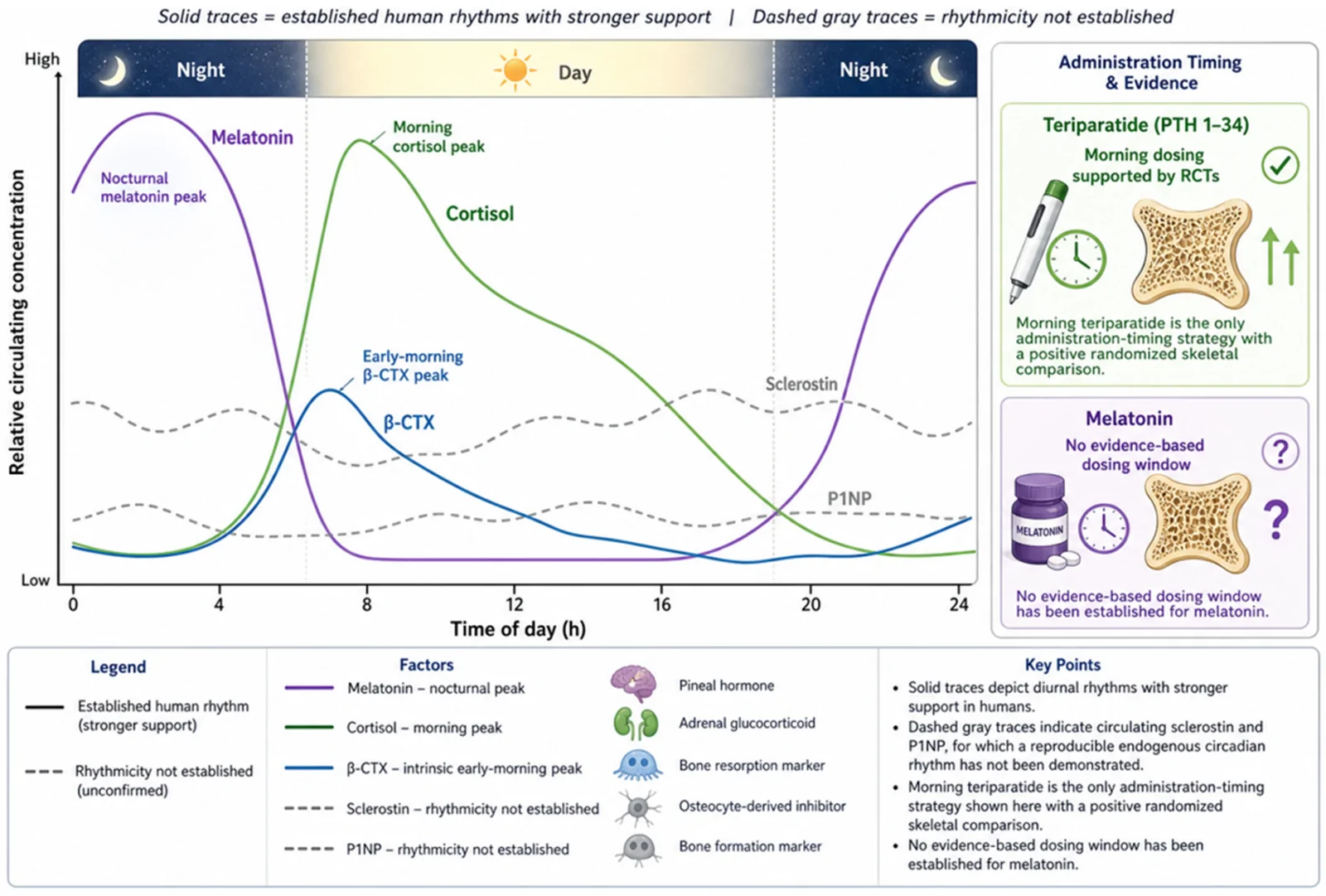
Diurnal rhythms of bone-remodeling factors: established and unconfirmed. Solid traces show human rhythms with stronger support, including nocturnal melatonin, morning cortisol, and the intrinsic early-morning β-CTX peak. Dashed gray traces indicate circulating sclerostin and P1NP, for which a reproducible endogenous circadian rhythm has not been demonstrated. Morning teriparatide is the only administration-timing strategy shown here with a positive randomized skeletal comparison; no evidence-based dosing window has been established for melatonin.

The evidence is uneven, and this distinction is load-bearing for any chronotherapeutic argument (Figure 2).

Established. Serum C-terminal telopeptide of type I collagen (β-CTX) exhibits an early-morning peak. This pattern has been reproduced across populations [53] and, under constant-routine conditions that minimize masking by sleep, posture, and feeding, has been shown to contain an endogenous circadian component with an acrophase near 03:00, approximately 5 h after dim-light melatonin onset [54]. In the same constant-routine study, circadian variation in bone-formation markers was limited, indicating stronger intrinsic rhythmicity of resorption than formation. In mice, Tnfsf11 and Tnfrsf11b transcription is regulated through E-box-dependent mechanisms, and their ratio varies in a Bmal1-dependent manner [8,32].

Not established. A reproducible circadian rhythm in circulating human sclerostin has not been demonstrated. In a study that sampled ten healthy men every 2 h for 24 h, Swanson and colleagues detected no significant rhythm in sclerostin (*p* = 0.99) or P1NP (*p* = 0.65), whereas CTX rhythmicity was confirmed (*p* < 0.001; peak, 05:30) and FGF-23 showed rhythmicity with variable acrophase [17]. These findings do not support the proposition that circulating sclerostin drives the daily rhythm of other bone-turnover markers.

Circadian regulation of Sost transcription is therefore supported in rodent models, whereas a corresponding rhythm in circulating human sclerostin protein remains unconfirmed; human rhythms in formation markers are also less consistently demonstrated than the rhythm in resorption. Chronotherapeutic arguments that depend on a human nocturnal sclerostin peak are therefore premature.

## 3. Molecular Mechanisms in the Osteoblast Lineage 

### 3.1. Receptor-Dependent: The MT2–Wnt/β-Catenin Axis 

**Figure 3:**
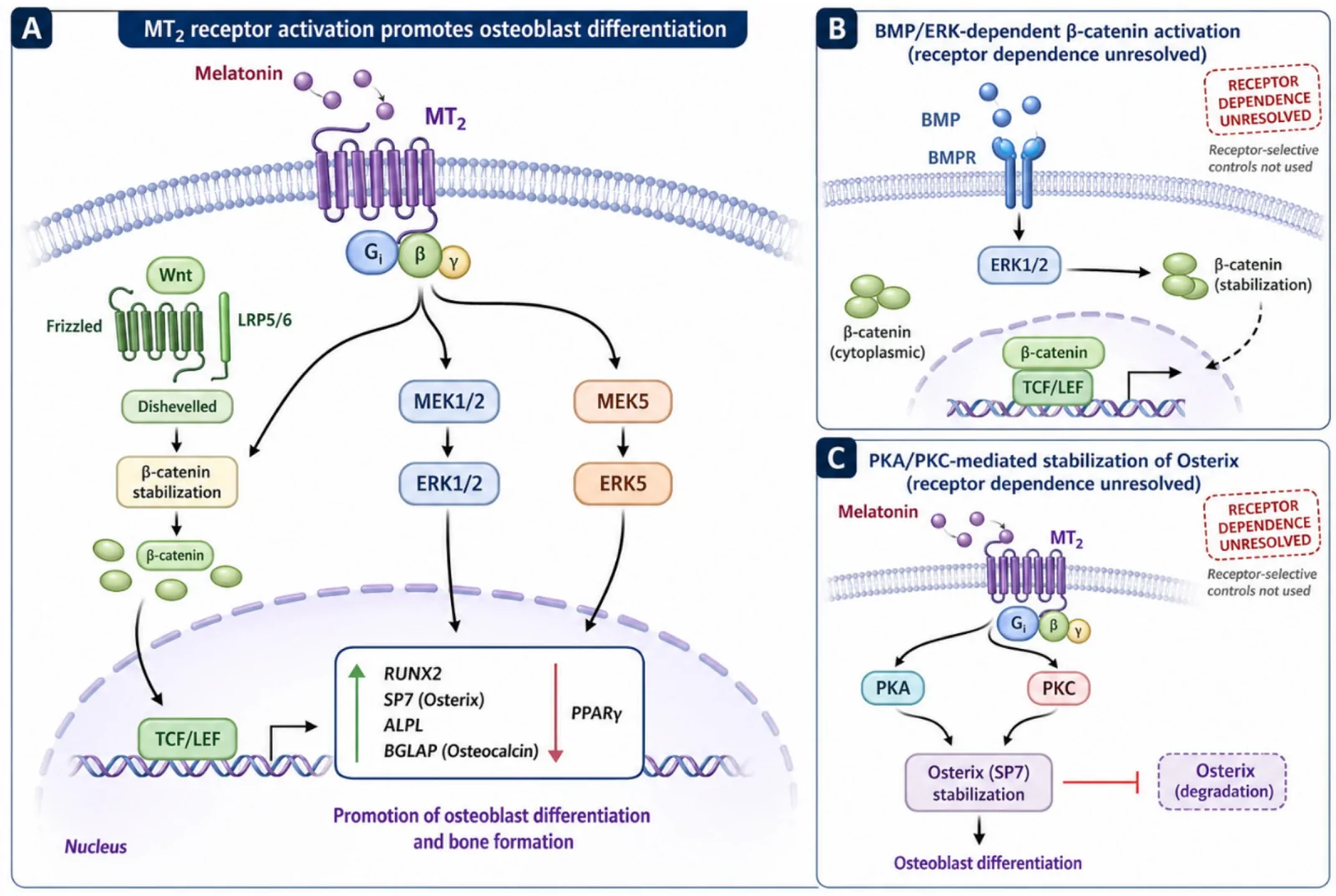
Receptor-dependent mechanisms of melatonin in the osteoblast lineage. MT_2_ activation promotes osteoblast differentiation through Wnt/β-catenin–TCF/LEF signalling and through the MEK1/2–ERK1/2 and MEK5–ERK5 cascades, increasing RUNX2, SP7, ALPL and BGLAP while suppressing PPARγ. Two further panels show BMP/ERK-dependent β-catenin activation and PKA/PKC-mediated stabilization of Osterix; both are annotated as unresolved for receptor dependence because receptor-selective controls were not used.

Canonical Wnt/β-catenin signaling is a central regulator of osteoblastogenesis. Wnt binding to Frizzled/LRP5/6 stabilizes cytosolic β-catenin, which translocates to the nucleus and, together with TCF/LEF, induces osteogenic genes including RUNX2, SP7, ALPL, and BGLAP [55] (Figure 3).

In bone-marrow-derived mesenchymal stromal cells, the MT2-selective antagonist 4-P-PDOT abolished melatonin-induced β-catenin activation and osteogenic differentiation, supporting MT2 involvement in that experimental system [20]. This antagonist experiment provides stronger evidence of receptor dependence than pathway changes observed after high-concentration melatonin alone; however, the 100 µM exposure used in the study is high pharmacological (Section 2.4).

**Figure 4:**
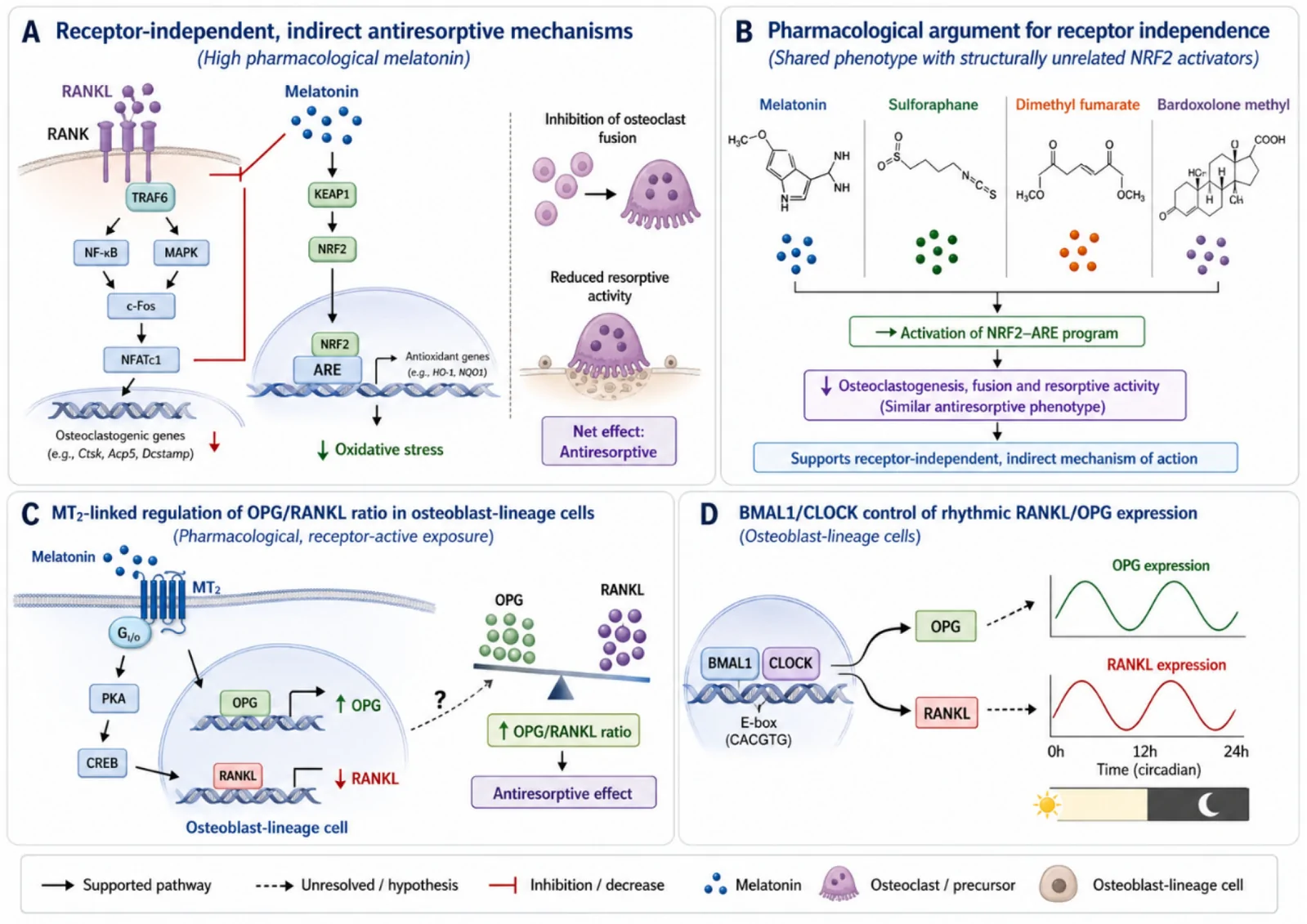
Cell-type-specific actions of melatonin in BMSCs and osteoblasts, classified by receptor dependence. MT_2_-linked Wnt/β-catenin and MEK1/2–MEK5 signaling is supported by receptor and pathway antagonism. BMP/ERK/JNK convergence was demonstrated at pharmacological exposure without receptor-null controls, whereas GSK-3β inhibition was reported at high pharmacological concentration without genetic confirmation. PKA/PKC-dependent stabilization of Osterix (SP7) is shown as a post-translational mechanism, but kinase inhibition does not establish receptor identity.

The same study reported inhibition of glycogen synthase kinase-3β (GSK-3β), stabilization of β-catenin, and induction of an lncRNA H19/miR-541-3p/adiponectin circuit that reinforced osteogenesis while suppressing adipogenic differentiation [20]. Because this non-coding-RNA mechanism derives from a single high-concentration study and has not been independently replicated or established in vivo, it is classified as exploratory in Table 2 and Figure 4.

### 3.2. Receptor-Dependent: MEK1/2 and MEK5 

The MEK1/2 and MEK5 cascades provide a second receptor-linked route. In human MSC/PBMC cocultures, melatonin-induced osteoblastogenesis was attenuated by 4-P-PDOT, the MEK1/2 inhibitor PD98059, and the MEK5 inhibitor BIX02189, with context-dependent changes in ERK1/2, ERK5, β1 integrin, GLUT4, and insulin receptor-β [13]. This study is informative because it used primary human cells and pharmacological receptor and pathway controls. The accompanying increase in RUNX2 and decrease in PPARγ place melatonin signaling at the osteogenic–adipogenic fate switch, a process relevant to age-related marrow adiposity (Figure 4).

### 3.3. Receptor Dependence Unresolved: BMP/ERK Convergence 

In MC3T3-E1 preosteoblasts, melatonin at 12.5–100 nM, with prominent effects at 50 nM, enhanced osteoblastic differentiation and mineralization, activated Wnt5a/b and β-catenin, and increased JNK and ERK phosphorylation. Inhibition of BMP signaling with noggin or ERK with PD98059 abolished melatonin-induced nuclear β-catenin translocation, positioning BMP and ERK upstream of Wnt/β-catenin in this model [21] (Figure 4).

The pathway evidence is informative, but the study did not use a melatonin-receptor antagonist, knockdown, or receptor-null control; receptor mediation therefore cannot be assigned. At 50 nM, the exposure is pharmacological but remains compatible with high-affinity receptor signaling, making direct antioxidant chemistry less compelling than in high pharmacological experiments. In addition, MC3T3-E1 is an immortalized murine cell line whose receptor complement and circadian properties may differ from those of primary human osteoblasts.

### 3.4. Post-Translational Control of Osterix (SP7) 

Osterix (SP7) is required for the progression of preosteoblasts to mature osteoblasts. At 1 µM, melatonin enhanced BMP-4-induced Osterix protein expression without increasing transcription by reducing ubiquitin–proteasome-mediated degradation, extending protein half-life, and increasing transcriptional activity at late osteogenic promoters. These changes were accompanied by greater alkaline phosphatase activity and mineralization. Inhibitors of protein kinase A (H89) and protein kinase C (Gö6976) blocked melatonin-induced Osterix phosphorylation and transcriptional activity, establishing PKA/PKC dependence of the reported response [22] (Figure 4).

Receptor dependence is therefore unresolved at a high pharmacological exposure of 1 µM (Section 2.4; Table 2, “Receptor dependence” and “Basis for attribution” columns). The mechanism is nevertheless notable because it operates post-translationally and provides a plausible explanation for the late-stage mineralization phenotype observed in the model.

### 3.5. Mixed Receptor Dependence: SIRT1/SIRT3 and Mitochondrial Protection 

Oxidative stress contributes to age-related and estrogen-deficiency bone loss. In H_2_O_2_-challenged MC3T3-E1 cells, melatonin increased alkaline phosphatase activity and mineralization, induced murine *Bmp2*, *Runx2*, and *Spp1*, activated SIRT1 and SIRT3, inhibited the pro-apoptotic p66Shc axis, reduced reactive oxygen species and malondialdehyde, increased superoxide dismutase activity, and reduced apoptosis [14]. In BMSCs obtained from osteoporotic donors, melatonin reduced intracellular oxidative stress and increased SOD2 and glutathione peroxidase; systemic melatonin also improved femoral microarchitecture in ovariectomized rats [15] (Figure 4).

The strongest causal evidence within this pathway comes from a loss-of-function experiment. In ovariectomized rats with periprosthetic bone loss, melatonin (50 mg/kg/day) increased mitochondrial SIRT3, reduced the acetyl-SOD2/SOD2 ratio, and increased periprosthetic bone mass; inhibition of SIRT3 abolished these effects, identifying SIRT3 as a required node in that model [16].

Receptor dependence in this pathway is therefore classified as mixed (Section 2.4; Table 2). The rodent regimen of 50 mg/kg/day is far above any dose given in a human bone trial (Section 8.1), so these data do not establish that clinically achievable oral exposure reproduces the antioxidant skeletal effects.

### 3.6. The ROS–KEAP1–NRF2 Axis as a Shared Antioxidant Node

The antioxidant actions described above—direct radical scavenging and SIRT1/SIRT3-mediated mitochondrial protection, with induction of superoxide dismutase and glutathione peroxidase as a downstream consequence—converge on one inducible transcriptional program, KEAP1–NRF2–ARE signaling.

Under basal conditions KEAP1 targets NRF2 for ubiquitination and proteasomal degradation. Electrophilic or oxidative modification of KEAP1 cysteines, or pharmacological disruption of the KEAP1–NRF2 interaction, stabilizes NRF2, which translocates to the nucleus and transactivates ARE-containing genes including those encoding superoxide dismutase, glutathione peroxidase, heme oxygenase-1 (HO-1), and NQO1. As set out in Section 2.2, this program is itself clock-controlled and feeds back on the clock [6,7].

In the osteoclast lineage the direction of effect is consistent. RANKL and M-CSF lower NRF2 and its downstream antioxidant enzymes during differentiation; *Nrf2*-deficient bone-marrow macrophages show heightened RANKL-induced ROS accumulation, enhanced MAPK and NF-κB activation, increased NFATc1, and increased osteoclast formation [56]. Conversely, pharmacological disruption of the KEAP1–NRF2 protein–protein interaction suppresses osteoclastogenesis in vitro and attenuates ovariectomy-induced bone loss in vivo [57]. NRF2 therefore provides the principal inducible counter-regulatory program to RANKL-associated ROS signaling.

Melatonin has been reported to activate NRF2/HO-1 signaling in osteoblastic cells, including in high-glucose models of diabetic bone fragility, and in BMSCs. Whether this occurs at receptor-active exposures, and whether it requires MT1 or MT2, has not been resolved by antagonist or genetic controls. Applying the criteria of Section 2.4, melatonin–NRF2 coupling in bone is therefore classified as unresolved rather than receptor-dependent, and it is entered as such in Table 2. Two qualifications are stated explicitly. First, the role of NRF2 in the osteoblast lineage is more context-dependent than in osteoclasts, and no blanket claim is made that NRF2 activation is uniformly osteoprotective. Second, the clock–NRF2 interlock described in Section 2.2 is established in fibroblasts, hepatocytes, and lung, not in bone; its skeletal operation is a hypothesis, and the factorial experiment that would test it is listed in Section 11.

### 3.7. ER Stress: PERK–eIF2α–ATF4

Under hyperglycemic conditions relevant to diabetes-associated bone fragility, 100 nM melatonin suppressed PERK–eIF2α–ATF4–CHOP signaling and reduced endoplasmic-reticulum-stress-associated apoptosis in hFOB1.19 osteoblasts, with pharmacological evidence implicating both MT1 and MT2 [12]. The finding is limited to one immortalized human cell line and has not been replicated in vivo; it should therefore be considered preliminary.

### 3.8. Clock-Intrinsic, Melatonin-Independent Effects

BMSCs isolated from Bmal1-null mice show impaired osteogenic differentiation in vitro, demonstrating a cell-autonomous requirement for BMAL1 in the osteoblast lineage [25]. In aged BMSCs, miR-142-3p represses BMAL1 and alters YAP-dependent osteogenesis; restoration of BMAL1 or antagomir-mediated inhibition of miR-142-3p improves molecular rhythmicity and osteogenic capacity, providing proof of concept for clock-modified cell therapy [58]. These findings concern intrinsic clock function and should not be interpreted as evidence of melatonin mediation.

## 4. Molecular Mechanisms in the Osteoclast Lineage

### 4.1. Direct Suppression of RANKL–NF-κB–MAPK–NFATc1 Signaling

**Figure 5:**
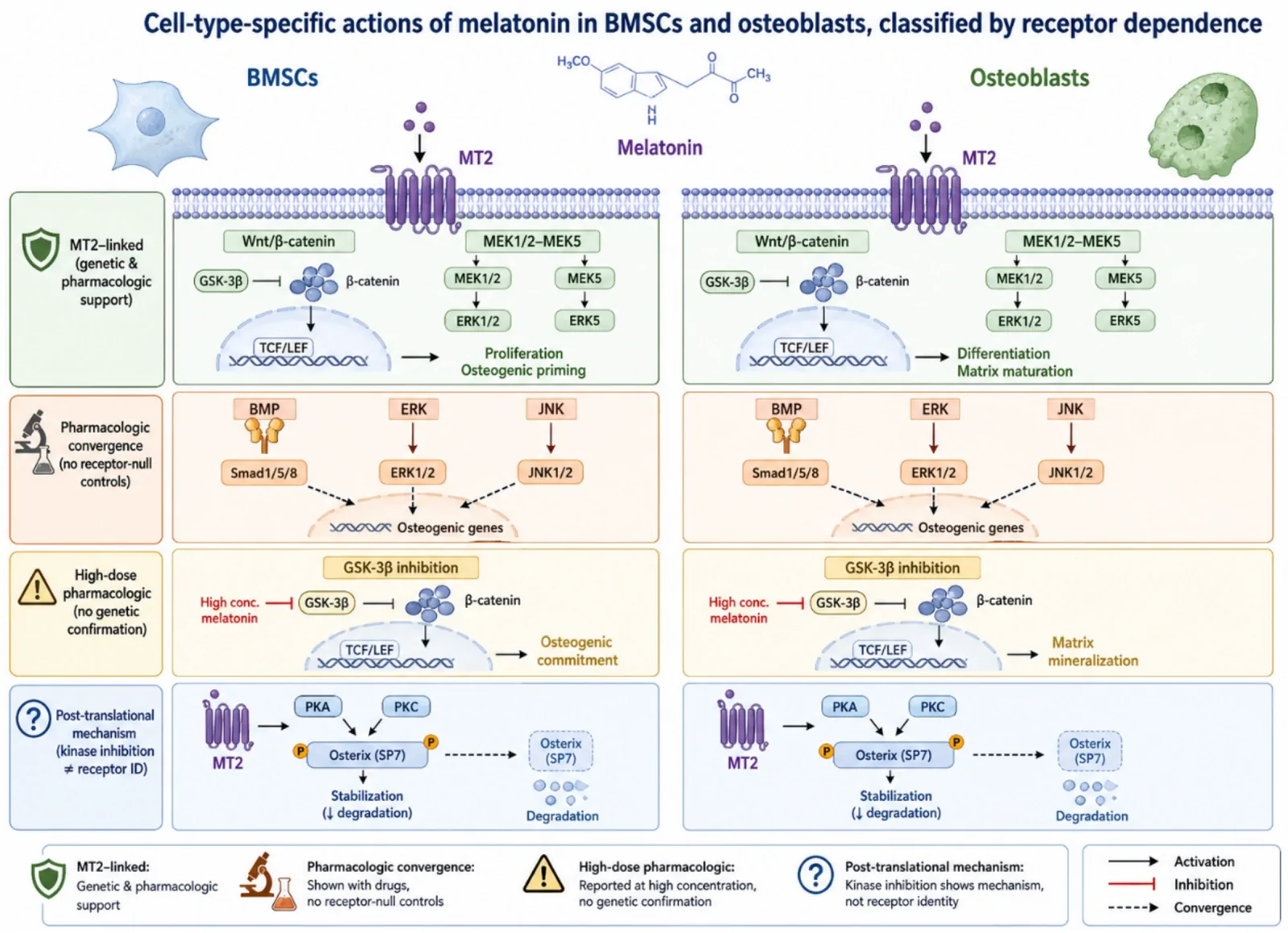
Receptor-independent and indirect antiresorptive mechanisms. At high pharmacological concentrations, melatonin suppresses RANKL–TRAF6–NF-κB/MAPK–c-Fos–NFATc1 signaling, lowers oxidative stress through induction of the KEAP1–NRF2–ARE program, and inhibits osteoclast fusion and resorptive activity. A separate panel shows the pharmacological argument for receptor independence, including the shared phenotype of structurally unrelated NRF2-activating compounds; a third shows MT_2_-linked regulation of the OPG/RANKL ratio in osteoblast-lineage cells at pharmacological, receptor-active exposure; and a fourth shows BMAL1/CLOCK control of rhythmic RANKL/OPG expression. Solid arrows indicate supported pathways; dashed arrows denote unresolved or hypothesis-generating relationships.

RANKL binding to RANK recruits TRAF6, activates canonical NF-κB and MAPKs (p38, JNK, and ERK) and c-Fos, and induces NFATc1, the master osteoclastogenic transcription factor [59]. Reactive oxygen species are important second messengers in RANKL signaling and contribute to propagation of NF-κB and MAPK pathways during osteoclast differentiation. The transcriptional program that opposes this ROS signal is KEAP1–NRF2–ARE (Section 3.6), which is downregulated by RANKL during differentiation and whose loss enhances osteoclastogenesis [56] (Figure 5).

At 100 µM, melatonin inhibited RANKL-induced NFATc1 nuclear translocation and reduced expression of the fusion-associated genes Dcstamp and Atp6v0d2, the latter encoding a vacuolar ATPase subunit required for acidification of the resorption lacuna. These changes impaired multinucleation and resorptive activity in RAW264.7 cells [23]. Melatonin also reduced RANKL-induced reactive oxygen species, TRAF6, and JNK signaling in related models [60].

### 4.2. Receptor-Independent Action: The Pharmacological Argument 

**Figure 6:**
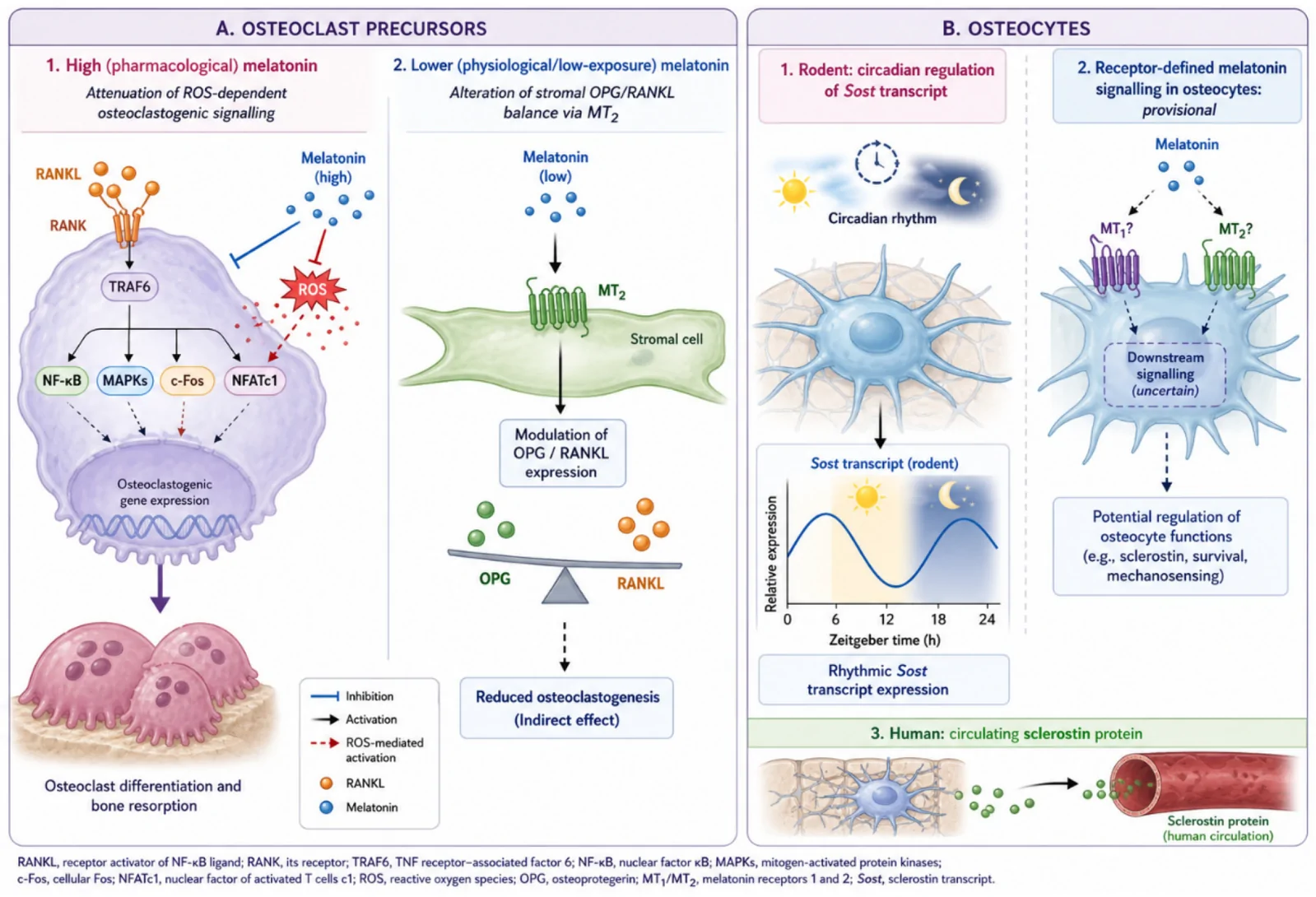
Cell-type-specific actions of melatonin in osteoclast precursors and osteocytes. In osteoclast precursors, RANKL activates TRAF6, NF-κB, MAPKs, c-Fos and NFATc1; high pharmacological melatonin attenuates ROS-dependent osteoclastogenic signaling, whereas a distinct lower-exposure pathway alters stromal OPG/RANKL through MT_2_. The osteocyte panel distinguishes rodent circadian regulation of the *SOST* transcript, and the provisional status of receptor-defined melatonin signaling in osteocytes; rodent *SOST* and human circulating sclerostin protein are distinguished typographically.

Two observations support a substantial receptor-independent component of the direct antiresorptive effect. First, MT1 and MT2 expressions decline during RANKL-driven osteoclast differentiation, reducing the machinery available for canonical signaling [24]. Second, direct suppression is generally observed at high pharmacological concentrations far above those required for high-affinity receptor binding. Scavenging of RANKL-induced reactive oxygen species, induction of the KEAP1–NRF2–ARE program and of superoxide dismutase, glutathione peroxidase, HO-1 and NQO1, and secondary attenuation of NF-κB and NFATc1 provide a coherent receptor-independent explanation [24,46] (Figure 6).

Chemically distinct antioxidant compounds provide independent support for this classification. 4-Methylcatechol, oroxylin A, and notopterol each suppress RANKL-induced osteoclastogenesis at micromolar concentrations with reduced ROS, attenuated MAPK/NF-κB signaling, and lower NFATc1 [61,62,63]. 4-Methylcatechol acts by binding KEAP1 and relieving its repression of NRF2 [61]. Notopterol likewise suppresses RANKL signaling and ROS-dependent osteoclastogenesis [63]. Collectively, these comparator data suggest that antiresorption at ≥10 µM can arise from shared ROS-lowering pathways rather than a melatonin-specific or MT-receptor-specific mechanism. This supports the classification in Section 2.4 and Table 2, in which direct high-concentration antiresorption is assigned primarily to receptor-independent mechanisms.

This distinction changes the translational hypothesis. If direct antiresorption requires micromolar tissue exposure, conventional nightly oral doses of 1–5 mg are unlikely to reproduce the high-concentration cell-culture effect. Representative 100 µM cell-culture exposures exceed the peak plasma concentration reached after a 100 mg oral dose by more than two orders of magnitude: reported Cmax after a 100 mg oral dose is of the order of 0.4 µM and decays within a few hours (Section 8.4) [64]. At clinically relevant exposure, receptor-mediated effects in osteoblast-lineage cells may therefore be more plausible than direct antioxidant suppression of mature osteoclasts. This proposition is not established clinically, but it is testable through adequately powered, dose-defined studies of β-CTX and P1NP and is more informative than further experiments confined to very high concentrations in immortalized cell lines (Figure 6).

### 4.3. Indirect Suppression via the OPG/RANKL Axis

OPG (TNFRSF11B), produced by osteoblasts, osteocytes, and stromal cells, acts as a decoy receptor for RANKL and prevents its interaction with RANK on osteoclast precursors. In BMSCs, 10–100 nM melatonin (pharmacological; receptor-active) increased OPG and reduced RANKL through an MT2-linked NF-κB mechanism, thereby suppressing BMMSC-mediated osteoclastogenesis in an indirect-contact coculture [19]. In human MSC/PBMC cocultures, melatonin also increased the OPG/RANKL ratio, but the antiosteoclastogenic response depended on coculture configuration [13].

This paracrine route is mechanistically distinct from the direct high-concentration effects described in Section 4.1 and Section 4.2. It is supported by receptor-level intervention at lower exposure and is therefore more compatible with physiological or near-physiological signaling. Its quantitative contribution to skeletal remodeling in vivo, however, remains to be established.

### 4.4. Circadian Control of RANKL Rhythmicity

BMAL1 and CLOCK regulate E-box-containing elements in the Tnfsf11 and Tnfrsf11b regulatory regions, and Bmal1 loss alters RANKL/OPG rhythmicity in mouse models [8,32]. Whether chronic circadian misalignment produces a cumulative increase in net resorption in humans remains a testable hypothesis rather than an established finding

## 5. Molecular Mechanisms in Osteocytes 

### 5.1. Sclerostin: Rodent Clock Control, Unconfirmed Human Rhythm 

Osteocytes constitute most cells in adult bone, form an interconnected lacuno-canalicular network within the mineralized matrix, and serve as principal skeletal mechanosensors that coordinate osteoblast and osteoclast activity [18]. They secrete sclerostin, encoded by *SOST*, a Wnt antagonist that binds LRP5/6 and inhibits β-catenin activation in adjacent osteoblasts. Loss or pharmacological inhibition of sclerostin activates Wnt/β-catenin signaling and increases bone mass, a mechanism exploited therapeutically by romosozumab (Figure 6).

Sost transcription is circadian in rodent models (Section 2.5), whereas the most detailed 24 h human profiling study found no rhythm in circulating human sclerostin protein [17]. Reports that melatonin suppresses the *SOST* transcript and restores Wnt signaling should therefore be regarded as preliminary; replicated evidence in primary human osteocytes and evidence of a physiologically meaningful change in circulating sclerostin protein are lacking.

### 5.2. Mechanotransduction and Survival

Osteocyte apoptosis in response to unloading, oxidative stress, or glucocorticoids releases pro-osteoclastogenic signals and initiates targeted remodeling. Direct evidence that melatonin protects osteocytes through SIRT1/SIRT3 is limited. High-concentration studies in osteoblast-lineage cells demonstrate SIRT1/SIRT3-associated mitochondrial protection, whereas Bmal1-null bone shows reduced viable osteocyte density [14,25]. Extrapolation of these findings to a receptor-defined mechanism in osteocytes should therefore be made cautiously.

FOXO transcription factors are plausible candidates because they coordinate antioxidant defense in skeletal cells and can interact with sirtuin pathways. Other stress-response pathways, including HIF-1α, protect osteoblasts from ROS-induced apoptosis in related models [65]. NRF2 belongs in the same list: it is the principal inducible antioxidant transcription factor in skeletal cells (Section 3.6), it is clock-controlled [6], and osteocytes are subject to the same oxidative challenges as the lineages in which it has been characterized. However, direct evidence that melatonin acts through FOXO1, HIF-1α or NRF2 in osteocytes is currently lacking; these pathways should be treated as research questions rather than established components of the circadian–melatonin axis.

### 5.3. Non-Coding RNA Circuits: Exploratory

miR-142-3p represses BMAL1 in aged BMSCs and contributes to loss of rhythmicity and osteogenic capacity [58]. By contrast, proposed roles for miR-34a, miR-21, or lncRNA MALAT1 in a melatonin/clock–osteocyte network have not been directly demonstrated in osteocytes. Such circuits should remain exploratory until validated in primary cells and in vivo.

## 6. The Bone Multicellular Unit: Cellular Composition, Circadian Coverage, and Tissue-Level Output 

**Figure 7:**
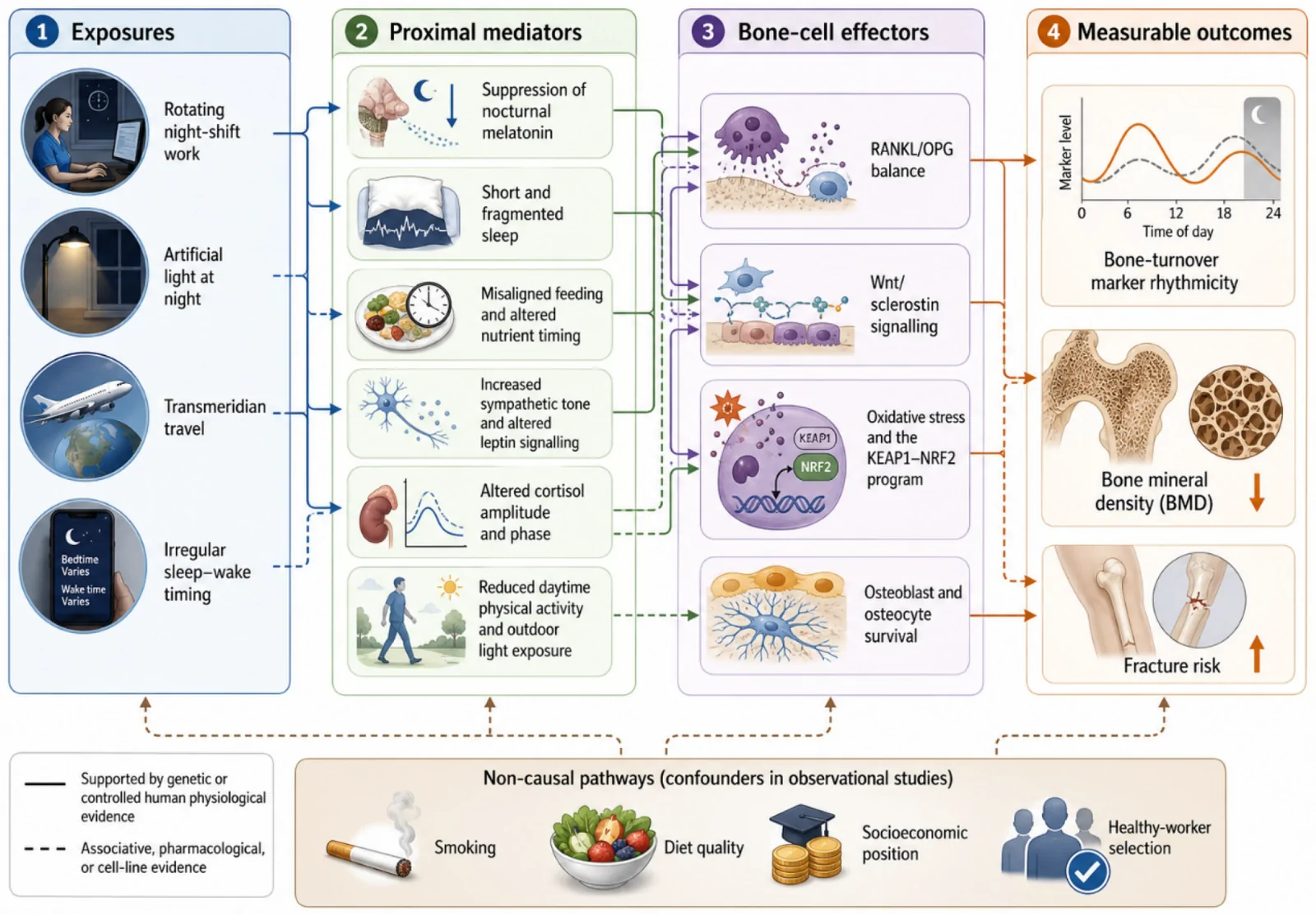
From bone-cell signaling to bone mineral density and fracture risk. A four-stage conceptual cascade: cellular signaling; bone multicellular unit behavior, resolved into activation frequency and remodeling balance; tissue-level architecture and mass, separated into trabecular and cortical compartments; and fracture risk. Melatonin-related mechanisms are positioned at their point of entry—MT_2_-linked anabolic signaling and SP7 stabilization act principally on remodeling balance, OPG/RANKL modulation principally on activation frequency. The constraints on extrapolation are annotated at the stage at which they apply: the small annual change in areal BMD produced by a change in balance, the contribution of the remodeling transient to early BMD change, differential trabecular and cortical response, and the absence of human-like intracortical Haversian remodeling in murine models. The figure carries the explicit annotation that no step distal to the cellular level has been demonstrated for melatonin in humans.

Section 3, Section 4 and Section 5 examine the three classical bone lineages. The bone multicellular unit (BMU), however, is a spatially organized remodeling compartment that also includes lining cells, osteomacs, vascular endothelium, marrow stromal and immune cells. This section maps circadian and melatonin evidence across that broader cellular context and identifies explicit evidence gaps (Table 4, Figure 7).

### 6.1. The BMU as an Organized Structure

Remodeling in adult bone proceeds within discrete BMUs in which resorption and formation are coupled in time and space [66]. A BMU is not simply an osteoclast followed by an osteoblast. It is covered by a canopy of bone lining cells that separates the remodeling compartment from the marrow; it is served by a dedicated capillary that delivers osteoclast and osteoblast precursors; it contains resident macrophages, termed osteomacs, that associate with the canopy and support osteoblast mineralization; and it is embedded in and directed by the osteocyte network, which initiates targeted remodeling. Marrow stromal, adipocytic, and immune populations contribute RANKL, OPG, and inflammatory mediators to the same compartment. Any cellular mechanism proposed to alter bone mass must act through this structure.

### 6.2. Circadian and Melatonin Coverage by Cell Type

Table 4 states, for each cell type in the BMU, its role, the circadian evidence, and the melatonin evidence. The evidence is concentrated in the three lineages around which Section 3, Section 4 and Section 5 are organized and is absent for bone lining cells, osteomacs, and skeletal endothelium.

### 6.3. Consequences for Interpretation

Three consequences follow. First, the Cre drivers used across the clock literature—*Prx1*, *Sp7*/*Osx*, *Col1a1*, *Dmp1*, *Ctsk* and *LysM*—label overlapping populations, and none isolates bone lining cells. Lineage attribution in that literature is therefore less clean than a table of genotypes suggests, and this qualification applies directly to the lineage-dependence argument made in Section 2.2. Second, systemic melatonin reaches every cell in the BMU, so a whole-organism skeletal phenotype cannot be assigned to one lineage without cell-restricted receptor deletion; this is the reason the corresponding experiments appear among the priorities in Section 11. Third, angiogenic–osteogenic coupling through type H (CD31^hi^EMCN^hi^) capillaries is an established determinant of bone formation that declines with age and after ovariectomy [68], so skeletal endothelium is a substantive omission rather than a peripheral one.

### 6.4. From Cellular Signaling to Tissue-Level Remodeling and Fracture Risk

Translation from cell-level signaling to bone mineral density and fracture risk depends on two BMU-level variables: the activation frequency with which new BMUs are initiated, and the remodeling balance—the difference between the volume formed and resorbed within each unit.

The mechanisms described above map onto these variables differently, and the distinction is testable. MT2-linked osteoblast anabolic signaling (Section 3.1 and Section 3.2) and SP7 stabilization (Section 3.4) would be expected to act principally on remodeling balance. Modulation of the OPG/RANKL ratio (Section 4.3) and clock control of *Tnfsf11*/*Tnfrsf11b* rhythmicity (Section 4.4) would act principally on activation frequency. The two produce different trajectories of bone-turnover markers and BMD over time—a balance effect raises formation markers with little early change in resorption markers, whereas a frequency effect lowers both—and distinguishing them requires a trial of only modest size, provided β-CTX and P1NP are measured serially.

Four constraints limit extrapolation from either variable to fracture. A change in remodeling balance produces only a small annual change in areal BMD. Part of any early BMD change reflects the remodeling transient reduction in the number of open resorption cavities—rather than a durable gain in bone matrix. Trabecular and cortical compartments respond differently, and areal DXA does not separate them. And murine models do not reproduce human intracortical Haversian remodeling, so preclinical architectural findings cannot be read directly as fracture-risk predictions. Figure 7 presents this as a four-stage cascade—cellular signaling, BMU behavior, tissue architecture and mass, and fracture risk—with the melatonin-related mechanisms positioned at their point of entry and with the explicit annotation that no step distal to the cellular level has been demonstrated for melatonin in humans.

## 7. Crosstalk with Systemic Endocrine and Behavioral Axes

### 7.1. Hypothalamic–Pituitary–Adrenal Axis

The hypothalamic–pituitary–adrenal axis generates a pronounced morning cortisol peak; chronic glucocorticoid excess increases local 11β-HSD1 activity, promotes RANKL expression, suppresses osteoblastogenesis, and accelerates osteocyte apoptosis [69]. Glucocorticoids can also convey circadian timing information to peripheral skeletal cells. These are two different exposures and should not be conflated. Rhythmic physiological cortisol acts as an entraining signal that conveys phase information to peripheral skeletal clocks and is a normal component of the timing system. Sustained pathological exposure, whether endogenous or exogenous, causes glucocorticoid-induced osteoporosis through the mechanisms above [70]. Loss of amplitude—a flattened cortisol rhythm without a change in mean exposure—is the pattern produced by chronodisruption, and its skeletal consequences have not been separately characterized.

### 7.2. Sex Steroids, and the Strength of the Menopause–Melatonin Link

Estrogen restrains bone turnover largely through the OPG/RANKL axis: it increases OPG production by osteoblast-lineage cells, reduces RANKL expression by osteoblasts, osteocytes, and marrow lymphocytes, and shortens osteoclast lifespan by promoting osteoclast apoptosis. Estrogen withdrawal therefore raises the RANKL/OPG ratio and increases both activation frequency and resorption depth, which is the dominant mechanism of early postmenopausal bone loss.

Established (moderately). Nocturnal melatonin amplitude declines with advancing age in humans. The supporting studies are predominantly cross-sectional and are subject to confounding by habitual light exposure, sleep timing, medication use, and assay differences, but the direction of effect is reasonably consistent.

Not established. That menopause independently attenuates the nocturnal melatonin signal after accounting for chronological age. The available human data are limited, inconsistent in direction, and derived largely from cross-sectional comparisons of pre- and postmenopausal women who also differ in age. We identified no adequately powered longitudinal study following the same women through the menopausal transition with repeated overnight or 24 h urinary sampling under controlled light conditions, which is the design that would be required.

Cannot address the question. Rodent ovariectomy experiments. Rodents do not undergo menopause; ovariectomy models estrogen deficiency but not the endocrine trajectory of the human transition. Moreover, as Section 2.2 notes, several widely used inbred strains carry nonfunctional *AANAT and ASMT alleles* and lack a rhythmic pineal melatonin signal altogether, so ovariectomy in those backgrounds cannot address an interaction between estrogen loss and melatonin decline even in principle.

The temporal convergence of estrogen loss with declining nocturnal melatonin is therefore, on current evidence, largely age-driven, with a menopause-specific component that is untested rather than demonstrated. The same convergence that motivates the hypothesis also makes the melatonin contribution difficult to identify in observational data, since age is a common cause of both. The longitudinal study required to separate them is listed in Section 11.

### 7.3. Leptin and Sympathetic Signaling

Adipokines and gut-derived signals are also circadian; among these, the leptin–sympathetic pathway is best characterized and underlies the high-bone-mass phenotype of Per-mutant mice [10].

The pathway is central rather than peripheral: leptin acting in the hypothalamus increases sympathetic outflow, and noradrenaline acting on β2-adrenergic receptors on osteoblast-lineage cells suppresses osteoblast proliferation and increases RANKL expression. The molecular clock in those cells gates this response, which is why Per1/Per2-mutant mice show high bone mass. This phenotype is informative about clock function and is not evidence about melatonin, which reinforces the distinction drawn in Section 2.2—an important point given that the strains concerned are frequently melatonin-deficient.

### 7.4. Feeding-Entrained and Metabolic Signals

Feeding is a dominant entrainer of peripheral clocks and carries several skeletally active signals. Insulin and the incretins are secreted rhythmically and act on osteoblast-lineage cells; the PTH–FGF-23 axis is diurnal, and human FGF-23 rhythmicity with a variable acrophase was demonstrated in the 24 h profiling study discussed in Section 2.5. *MTNR1B* is among the most robustly replicated glycemic loci, and the rs10830963 G allele is associated with elevated fasting glucose and impaired early insulin secretion [71]; yet no skeletal effect of that variant has been demonstrated. Melatonin therefore has a well-established metabolic action mediated by a receptor whose skeletal role is separately established, without the two having been shown to connect. This distinction is relevant to both candidate stratification (Section 10) and safety (Section 8.4).

### 7.5. Sleep as a Distinct Pathway

Melatonin is a hormonal signal; sleep is a behavioral output. Sleep duration and quality have independently been associated with skeletal outcomes [72]. Actigraphic sleep patterns have been associated with bone turnover and BMD in young adults [73], while short or fragmented sleep has been associated with bone measures in older men [74] and BMD in older postmenopausal women [75], although findings are not uniform across cohorts. Sleep disruption may also mediate part of the shift-work association discussed in Section 8.3. Because melatonin improves sleep, no existing trial can cleanly separate direct skeletal effects from indirect effects mediated by sleep, activity, or fall risk. At high chronic doses, this distinction also becomes a safety consideration (Section 8.4).

### 7.6. Integration: Melatonin’s Place in the Network

Relative to sex steroids, glucocorticoids, and the PTH–FGF-23 axis, melatonin is a low-amplitude modulatory input to bone. It is not an objection to the melatonin hypothesis; it is a constraint on the effect size any melatonin intervention should be expected to produce, and it is consistent with the small and inconsistent effects reported in the five trials summarized in Table 3. Melatonin is best understood as a timing signal superimposed on stronger determinants of remodeling, and its skeletal effects are therefore expected to be modulatory rather than dominant.

The concurrent changes in estrogen, melatonin, and turnover rhythms at menopause are sometimes described as a compounding “triple hit.” Their temporal convergence is plausible, but additive or multiplicative interaction has not been demonstrated. The relationship should therefore be framed as a hypothesis rather than an established integrated mechanism. On current evidence the convergence is attributable principally to age (Section 7.2).

## 8. Preclinical and Clinical Evidence

### 8.1. Preclinical Evidence

Across gonadectomy and ovariectomized rodent models, exogenous melatonin has generally preserved trabecular architecture, increased osteoblast-associated indices, reduced osteoclast activity, or improved biomechanical properties [11,16,57]. Local or sustained-delivery studies further suggest that exposure kinetics can influence osseointegration and bone formation, although formulation and model heterogeneity preclude a unified quantitative conclusion. In aged models, melatonin has also improved BMSC osteogenic capacity in association with SIRT1-linked antioxidant defense [15].

Two translational caveats are important. First, preclinical doses are commonly much higher than human trial doses on a body-weight basis, and species differences in melatonin clearance make direct allometric extrapolation unreliable. Quantitatively, the 50 mg/kg/day rat regimen in the SIRT3 experiment corresponds to a body-surface-area-adjusted human equivalent of roughly 8 mg/kg, or about 550–600 mg daily for a 70 kg adult—approximately thirty times the 20 mg used in the highest-dose human trial (Table 3; Section 8.4). Second, mice are nocturnal, so the phase relationships among activity, feeding, and melatonin differ from those in diurnal humans; moreover, murine cortical bone does not reproduce the extent and organization of intracortical Haversian remodeling found in adult humans. Rodent findings should therefore be considered mechanistically informative rather than directly dose-translatable.

### 8.2. Clinical Evidence: Monotherapy and Combination Therapy Considered Separately 

Five small, randomized trials have reported skeletal or musculoskeletal outcomes. Because several combined melatonin with other active agents or osteogenic loading, their results cannot be pooled as a single estimate of melatonin efficacy. Table 3 therefore separates monotherapy from combination or factorial designs and summarizes study-level limitations (Table 3).

Melatonin monotherapy. MelaOst randomized 81 postmenopausal women with osteopenia to melatonin 1 mg (*n* = 20), melatonin 3 mg (*n* = 20), or placebo (*n* = 41) for 12 months, with bone mineral density (BMD) assessed by DXA, QCT, and HR-pQCT [26]. A small femoral-neck areal BMD difference favored melatonin and reached statistical significance in the 3 mg group; volumetric spine BMD also improved at the higher dose, whereas bone-turnover markers did not change significantly. The trial provides a preliminary densitometric signal but had only 20 participants in each active arm and assessed multiple skeletal sites, so the finding requires independent replication.

MOPS randomized 18 perimenopausal women aged 45–54 years to melatonin 3 mg (*n* = 13) or placebo (*n* = 5) nightly for 6 months, with calcaneal ultrasound and serial osteocalcin and NTX measurements [27]. No significant change was detected in bone density or either individual turnover marker. Quality-of-life measures and menstrual regularity improved, and the authors noted a directional change in the osteocalcin/NTX ratio. For skeletal outcomes, however, MOPS was a null and severely underpowered trial; it did not assess femoral-neck BMD by DXA.

Melatonin in combination. MOTS randomized 22 postmenopausal women with osteopenia to a four-component nightly combination of melatonin 5 mg, strontium citrate 450 mg, vitamin D_3_ 2000 IU, and vitamin K_2_ (MK-7) 60 µg (*n* = 11) or placebo (*n* = 11) for 12 months [28]. Relative to placebo, lumbar-spine BMD increased by 4.3% and left femoral-neck BMD by 2.2%; P1NP increased, the CTX/P1NP ratio decreased, and total hip BMD showed a nonsignificant upward trend (*p* = 0.069). Accompanying coculture experiments showed increased osteoblastogenesis, reduced osteoclastogenesis, increased OPG, and reduced RANKL. Because strontium incorporation inflates DXA-derived areal BMD independently of an equivalent gain in bone mass, the melatonin contribution cannot be isolated and MOTS should not be regarded as a replication of monotherapy (Table 3).

MelaOstrong was a 2 × 2 factorial pilot that analyzed 19 postmenopausal women with osteopenia and compared mock or osteogenic loading with melatonin 5 mg or placebo for 12 months [29]. Functional testing and patient-reported outcomes were emphasized, although DXA was also performed. Timed-up-and-go and sit-to-stand performance worsened in the mock-loading/placebo group but not in the intervention groups, and exploratory correlations with turnover markers were reported. No clear between-group BMD benefit attributable to melatonin was demonstrated. With group sizes of *n* = 4–6, the study cannot establish an effect on bone density, bone strength, or fracture risk (Table 3).

Melatonin in secondary osteoporosis. A randomized trial enrolled 41 patients with iron-overloaded thalassemia, low BMD (Z-score < −2), and ferritin > 500 µg/L and assigned them to melatonin 20 mg nightly (*n* = 21) or placebo (*n* = 20) for 12 months [30]. Lumbar-spine BMD did not differ significantly between groups at 12 months (*p* = 0.069). Within the melatonin group, lumbar-spine BMD increased from baseline (*p* = 0.029), and P1NP and malondialdehyde decreased at 6 months; back-pain scores also improved.

The randomized between-group comparison is the appropriate efficacy test and was not statistically significant. A within-group change in one arm does not establish a treatment effect. The reductions in oxidative-stress markers are mechanistically interesting but remain biomarker findings, and the iron-overloaded thalassemia population cannot be generalized directly to primary postmenopausal osteoporosis.

Synthesis. The clinical literature comprises very small samples, heterogeneous populations, doses of 1–20 mg, and differing interventions and measurement methods, with the study-level limitations set out in Table 3. No trial was powered for fracture prevention, and none enrolled a population with established primary osteoporosis; the efficacy of melatonin for osteoporosis therefore remains unestablished.

### 8.3. Epidemiology: Shift Work and Chronodisruption

**Figure 8:**
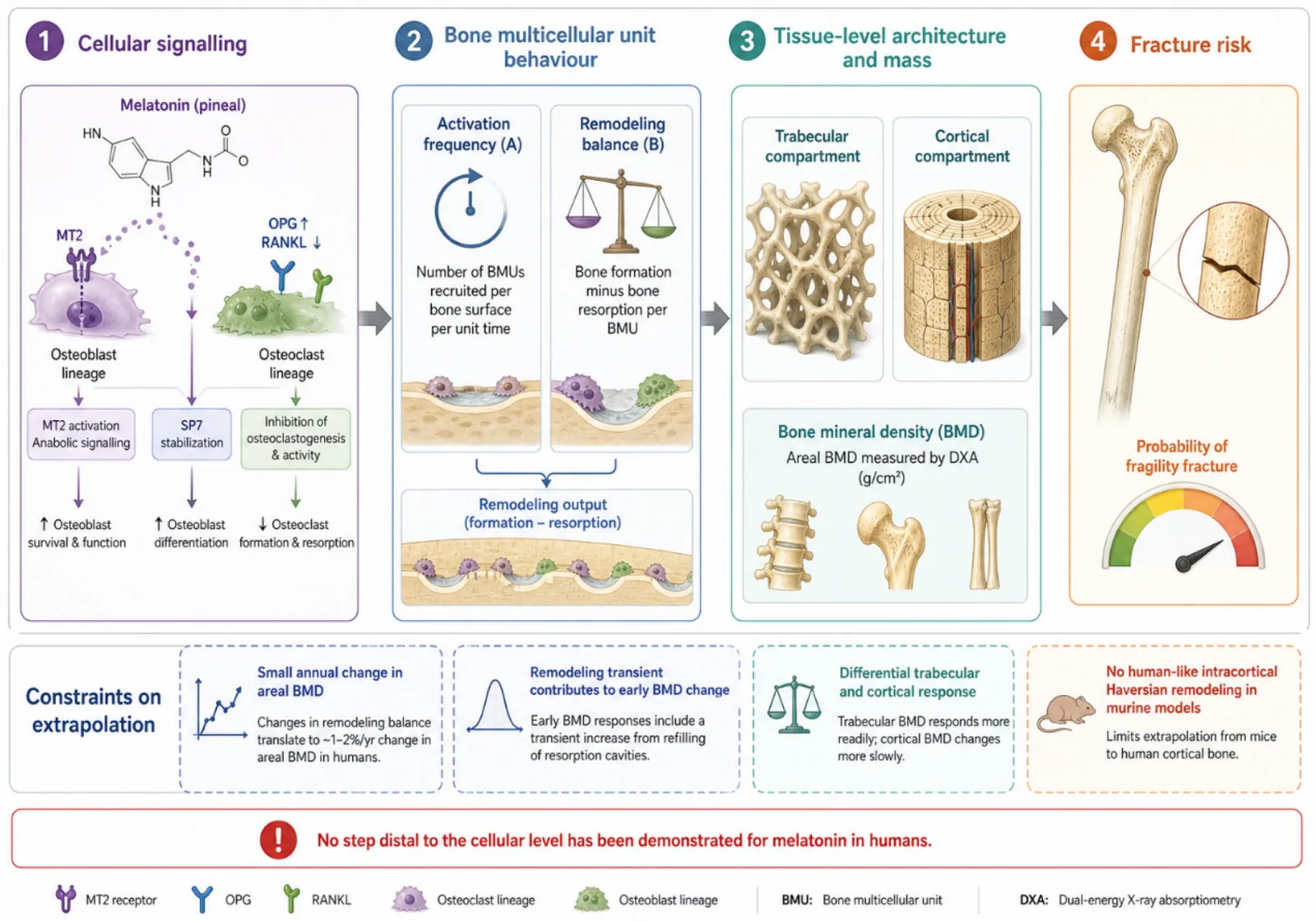
Pathways linking circadian disruption to skeletal outcomes. Four columns: (i) exposures—rotating night-shift work, artificial light at night, transmeridian travel, and irregular sleep–wake timing; (ii) proximal mediators—suppression of nocturnal melatonin, short and fragmented sleep, misaligned feeding and altered nutrient timing, increased sympathetic tone and altered leptin signaling, altered cortisol amplitude and phase, and reduced daytime physical activity and outdoor light exposure; (iii) bone-cell effectors—RANKL/OPG balance, Wnt/sclerostin signaling, oxidative stress and the KEAP1–NRF2 program, and osteoblast and osteocyte survival; and (iv) measurable outcomes—bone-turnover marker rhythmicity, BMD, and fracture. Consistent with Figure 1, Figure 2, Figure 3, Figure 4, Figure 5, Figure 6 and Figure 7, solid connectors denote relationships supported by genetic or controlled human physiological evidence and dashed connectors denote associative, pharmacological, or cell-line evidence. A separate shaded channel marks the non-causal pathways—smoking, diet quality, socioeconomic position, and healthy-worker selection—that confound the observational literature, so that the figure does not overstate the causal claim.

Before the estimates are reviewed, it is worth displaying the pathways that make causal attribution difficult. Figure 8 sets out the exposures (rotating night-shift work, artificial light at night, transmeridian travel, irregular sleep–wake timing), the proximal mediators through which they could act (suppression of nocturnal melatonin, short and fragmented sleep, misaligned feeding, increased sympathetic tone and altered leptin signaling, altered cortisol amplitude and phase, and reduced daytime activity and outdoor light), the bone-cell effectors (RANKL/OPG balance, Wnt/sclerostin signaling, oxidative stress, osteoblast and osteocyte survival), and the measurable outcomes. A separate shaded channel marks the non-causal pathways—smoking, diet quality, socioeconomic position, and healthy-worker selection—that confound this literature. Melatonin is one mediator among several, and the figure is drawn so that the review does not overstate the causal claim (Table 5, Figure 8).

In the Nurses’ Health Study, at least 20 years of rotating night-shift work was associated with a higher risk of wrist and hip fracture over 8 years compared with no night-shift work (RR 1.37, 95% CI 1.04–1.80); the strongest association occurred among women with body mass index < 24 kg/m^2^ who had never used hormone-replacement therapy (RR 2.36, 95% CI 1.33–4.20) [76]. The association was not apparent over 12 years after a single baseline exposure assessment, and residual confounding could not be excluded.

A more recent UK Biobank analysis included 276,774 currently employed participants and 75,120 participants with lifetime occupational histories and documented 5906 incident osteoporosis events [77]. Usual night-shift work was associated with incident osteoporosis (HR 1.29, 95% CI 1.12–1.50). In lifetime-schedule analyses, 3–8-night shifts per month were associated with osteoporosis (OR 1.38, 95% CI 1.11–1.72), whereas a duration exceeding 10 years was not (OR 1.21, 95% CI 0.92–1.58). An association with coded osteoporosis-related pathological fracture was also reported (HR 1.88, 95% CI 1.20–3.94), and no interaction with genetic susceptibility was detected.

Three distinctions are important. The HR of 1.88 pertains to coded pathological fracture, not incident osteoporosis, for which the estimate was 1.29. Its confidence interval is wide, indicating imprecision, and “pathological fracture” in administrative coding is not synonymous with an osteoporotic fragility fracture. Finally, the absence of statistical interaction with polygenic risk indicates no detected effect modification; it does not establish freedom from confounding.

Cross-sectional studies and reviews generally report lower BMD or altered turnover markers in shift workers, but exposure definitions vary and confounding by physical activity, sleep duration, diet, smoking, and socioeconomic position remains substantial [72,78]. Healthy-worker selection and reverse causation are also plausible. Prospective studies with repeated exposure assessment and causal-inference approaches, including appropriately instrumented Mendelian-randomization analyses, would strengthen this evidence base.

### 8.4. Attainable Tissue Exposure and the Safety of Long-Term High-Dose Melatonin (Table 6)

This section separates pharmacokinetic feasibility from the safety evidence for sustained high-dose use. Physiological nocturnal concentrations are 0.26–0.65 nM (Section 2.4). In a systematic review of human pharmacokinetic studies, reported Cmax after a 100 mg oral dose was of the order of 0.4 µM and decayed within a few hours, against an absolute oral bioavailability of approximately 9–33% and wide interindividual variation in the absorbed fraction [64]. Sustained exposure at or above 1 µM is therefore not attainable by conventional oral administration; representative 100 µM experiments exceed the peak concentration after a 100 mg dose by more than two orders of magnitude. The same exposure gap is evident in the preclinical literature (Section 8.1).

The liabilities identified here are those that matter specifically for a skeletal indication. The controlled literature at doses of 10 mg and above shows an excess of drowsiness, headache and dizziness without a detectable excess of serious adverse events, but it is thin: only four trials met prespecified low risk-of-bias criteria and more than a third did not report adverse events at all [79]. Two liabilities deserve particular emphasis.

First, exogenous melatonin impairs glucose tolerance and early insulin secretion, and in a randomized crossover study the effect was confined to carriers of the *MTNR1B G* allele at rs10830963 [80]—the same variant that Table 7 lists as a candidate stratification marker. The variant that might identify responders is therefore also the variant that identifies those most susceptible to a metabolic adverse effect. Because dysglycemia is prevalent in the target population and evening dosing coincides with any late carbohydrate intake, this is not a theoretical concern.

Second, because melatonin improves sleep and no trial has partitioned direct from indirect skeletal effects (Section 7.5), sedation at high chronic doses becomes a potential liability and not only a confounder: most fragility fractures follow a fall. Table 6 summarizes these domains together with the duration, formulation, and drug-interaction considerations.

These data do not imply that conventional low-dose melatonin is unsafe. Rather, the sustained micromolar exposure required by many receptor-independent mechanisms lies outside the range adequately characterized in humans, so long-term safety at such exposure remains unknown.

## 9. Chronotherapy: What Is and Is Not Supported

Chronotherapy—aligning treatment timing with endogenous biological rhythms—is a legitimate and testable concept in bone because human resorption is rhythmic and some therapeutic targets show time-dependent biology. The evidence is nevertheless agent-specific and should not be generalized across treatments.

Teriparatide: supported by randomized evidence. Fifty women with established postmenopausal osteoporosis were randomized to teriparatide 20 µg each morning or evening for 12 months. Lumbar-spine BMD increased in both groups, with a significantly greater increase after morning administration [85]. Earlier work showed that evening teriparatide preserved the nocturnal CTX rhythm, whereas morning administration attenuated the nocturnal CTX peak [86]. This is the strongest randomized chronotherapeutic signal in the skeletal literature, although the trial was modest in size and open label. A published protocol is evaluating 08:00 versus 20:00 administration over 12 weeks [87].

Melatonin: physiological rationale without skeletal timing evidence. Evening administration is consistent with its chronobiotic use and was adopted in the available trials. This convention is not evidence of skeletal optimization: no trial has compared different melatonin administration times using a bone endpoint, and no trial has individualized dosing to dim-light melatonin onset.

Bisphosphonates: pharmacokinetics rather than chronobiology. Morning fasting administration of oral bisphosphonates is dictated by absorption requirements and product labeling. It should not be interpreted as evidence that circadian targeting of osteoclasts improves efficacy.

Thus, skeletal chronotherapy currently has one positive randomized timing comparison, for teriparatide. Applying the same principle to melatonin remains a biologically plausible but untested hypothesis.

## 10. Toward a Research Framework 

A clinical decision algorithm is not yet justified: no skeletal trial has tested chronotype- or genotype-guided melatonin dosing, isolated interactions with approved osteoporosis drugs, or validated biomarkers that predict skeletal response.

Table 7 summarizes candidate stratification variables and their current validation status, and assigns each a research priority (high, intermediate, or exploratory) aligned with Section 11.1. Several points warrant emphasis.

Chronotype questionnaires, urinary 6-sulfatoxymelatonin, dim-light melatonin onset, cortisol profiles, and bone-turnover rhythms are measurable. It is plausible that participants with documented chronodisruption or low endogenous melatonin could respond differently, but this has not been tested. Biomarker-enriched trials would therefore be hypothesis-driven research rather than implementation studies.

The MTNR1B variant rs10830963 is robustly associated with fasting glucose and type 2 diabetes [71]. We identified no evidence linking it to BMD, fracture, or skeletal response to melatonin. Table 7 therefore retains it only as an unvalidated candidate variable, and Section 8.4 notes that it also marks susceptibility to melatonin-induced impairment of glucose tolerance [80].

Nonpharmacological circadian optimization—regular sleep–wake schedules, reduced nocturnal light, and appropriately timed morning bright light—is low risk and supported for circadian outcomes generally, but a skeletal benefit has not been demonstrated; reducing prolonged night-shift exposure may be reasonable on occupational-health grounds, and any bone-specific recommendation rests on observational evidence alone (Section 8.3, Figure 8).

## 11. Knowledge Gaps and Priority Experiments

### 11.1. Priorities for the Next Few Years

Four priorities are most likely to change interpretation of the field within the next few years.

(1) Human tissue pharmacokinetics. Simultaneous measurement of melatonin in plasma, bone, and bone marrow after oral dosing determines whether the concentrations required for the receptor-independent mechanisms are attainable at any tolerable dose. This is the single measurement that most constrains translational interpretation (Section 8.4).
(2) Receptor-specific in vivo dose response. A systematic comparison in wild-type and Mtnr1b-null animals across both anabolic and antiresorptive outcomes partitions receptor-dependent from receptor-independent efficacy.
(3) An adequately powered, preregistered monotherapy trial in established primary osteoporosis, with prespecified densitometric and bone-turnover endpoints, prospective structured adverse-event capture, and assessment of glucose tolerance rather than an assumption that safety is established by the sleep literature.
(4) Adequately powered constant-routine profiling of sclerostin and bone-formation markers in women and older adults, to determine whether the rodent Sost rhythm has a human circulating correlate.

### 11.2. Additional Priorities

Additional questions remain important but are less likely, individually, to change the overall interpretation of the field.

(1) Fracture endpoints. A fracture-outcome trial should be contemplated only after a reproducible effect on validated intermediate skeletal outcomes has been demonstrated and an appropriate sample-size calculation has been performed.
(2) Receptor-subtype-selective ligands. The nonredundant roles of MT1 and MT2 in primary human bone cells remain incompletely characterized. Selective agonists and antagonists would permit mechanistic dissection and may ultimately provide greater tissue and pathway specificity.
(3) Osteocyte signal transduction. The mechanism by which systemic melatonin might alter osteocyte-derived RANKL or sclerostin requires direct investigation in primary osteocytes and in vivo. Candidate sirtuin, NRF2, and FOXO pathways should be tested rather than assumed.
(4) Epigenetic consequences of chronodisruption. Whether chronic misalignment produces persistent DNA-methylation or histone changes at clock and osteogenic loci, and whether these changes are reversible, remains unknown and could help explain prolonged effects after exposure ends.
(5) Combination and interaction studies. Interactions with bisphosphonates, denosumab, and romosozumab have not been defined. Proposed synergy with romosozumab is speculative, particularly because a human circulating sclerostin rhythm has not been established.
(6) Sex- and age-specific effects. Human data derive predominantly from peri- and postmenopausal women; skeletal effects in older men and in younger populations remain largely unstudied.
(7) Causal inference in epidemiology. Repeatedly assessed prospective cohorts, negative-control analyses, and carefully selected genetic instruments for chronotype or sleep traits could help address residual confounding in the shift-work literature.
(8) Circadian therapeutics beyond melatonin. REV-ERB ligands, CRY stabilizers, and BMAL1-directed approaches are useful experimental tools, but their skeletal selectivity, efficacy, and safety have not been established.
(9) Partitioning the antioxidant effect. A factorial experiment using *Nrf2*-null and wild-type bone-marrow macrophages and osteoblasts, with and without MT2 antagonism, would separate melatonin’s skeletal antioxidant effect into NRF2-dependent and receptor-dependent components. This is a well-posed and inexpensive experiment that follows directly from the argument in Section 3.6.
(10) Cell-restricted deletion of Mtnr1b in endothelial and myeloid compartments, to test whether skeletal melatonin effects are confined to the osteoblast lineage (Section 6.3).
(11) Characterization of clock function and melatonin responsiveness in primary skeletal endothelial cells and osteomacs, the two largest coverage gaps identified in Table 4.
(12) A longitudinal human study of nocturnal melatonin amplitude across the menopausal transition, using repeated dim-light melatonin onset or overnight urinary 6-sulfatoxymelatonin measurement under controlled light conditions. This is tractable and inexpensive relative to the other priorities listed here and would resolve the question raised in Section 7.2.

## 12. Limitations

The mechanistic evidence base relies heavily on immortalized cell lines and on exposures far above physiological plasma concentrations; the specific lines, the exposure categories, and the attribution criteria are set out in Section 2.4 and Table 2 and are not restated here. Rodent-to-human translation is further complicated by high preclinical doses, faster clearance, species differences in receptor pharmacology, the inverted phase relationship between activity and melatonin in nocturnal animals, limited correspondence of murine cortical remodeling to adult human Haversian remodeling, and melatonin deficiency in commonly used inbred strains.

The mechanistic literature is also concentrated in three lineages—osteoblasts, osteoclasts, and osteocytes—and the coverage of this review necessarily reflects that concentration. As Table 4 records, no dedicated circadian or melatonin study was identified for bone lining cells, osteomacs, or skeletal endothelium, so the account given of the BMU in Section 6 is a map of where evidence exists rather than a synthesis of evidence about every component.

Human trials are small (largest *n* = 81), short (≤12 months), and heterogeneous in population, dose (1–20 mg), intervention composition, and densitometric method. Several were null on their randomized skeletal comparison, and the MOTS trial [28] cannot isolate melatonin and is complicated by strontium-related DXA effects. Publication bias is possible because much of the clinical literature arises from a small number of groups. Melatonin can also improve sleep, making it difficult to separate a direct skeletal effect from indirect changes in sleep quality, activity, or fall risk; no trial was specifically designed to partition these pathways (Section 7.5). No skeletal trial exceeded 12 months, and long-term safety at doses above those tested is unestablished rather than favorable (Section 8.4).

## 13. Conclusions

Melatonin is a biologically plausible modulator of skeletal remodeling. Receptor-dependent evidence supports MT2-linked osteoblast differentiation through Wnt/β-catenin and MEK signaling, SP7 stabilization, and indirect regulation of osteoclastogenesis through the OPG/RANKL axis. Direct antiosteoclastic and antioxidant effects, however, are reported mainly at pharmacological or high-pharmacological concentrations and are more consistent with receptor-independent chemistry converging on ROS–KEAP1–NRF2 signaling.

Clinical evidence remains insufficient for therapeutic recommendation. The five randomized trials are small and heterogeneous, none was powered for fracture prevention, and none established efficacy in primary osteoporosis. Chronotype-, biomarker-, or genotype-guided dosing therefore remains investigational, while high-dose regimens intended to reproduce receptor-independent effects lack both pharmacokinetic feasibility and long-term safety evidence.

For current practice, melatonin should not replace indicated antiresorptive or anabolic therapy. Circadian hygiene may be reasonable for general health, but a skeletal benefit has not been demonstrated; prolonged night-shift exposure should prompt standard fracture-risk assessment rather than melatonin treatment. Future trials should test melatonin monotherapy in established osteoporosis with prespecified BMD, bone-turnover, exposure, and safety endpoints. The most informative next steps are human bone and marrow pharmacokinetic studies, receptor-specific in vivo dose–response experiments, and an adequately powered monotherapy trial. Together, these studies would determine whether the circadian–melatonin axis is a clinically actionable target or primarily an explanatory framework.

## Figures and Tables

**Table 1:** Core molecular components of the circadian–melatonin axis and their principal skeletal actions, with evidence tier.

Component	Class/Function	Principal Skeletal Action	Species/System	Ref.
BMAL1/CLOCK	Core clock heterodimer, E-box transactivator	Cell-autonomous positive regulator of osteoblast differentiation; sets phase of Tnfsf11/Tnfrsf11b and Sost oscillation	Mouse, global and conditional knockout	[5,6,7]
BMAL1 (osteoclast-specific)	Lineage-restricted deletion (Ctsk-Cre)	Impaired osteoclastogenesis; reduced Acp5, Nfatc1; high bone mass	Mouse	[8]
CLOCK	Core clock heterodimer partner	Clock-mutant mice show reduced bone density and increased apoptosis via PDIA3	Mouse	[9]
PER1/PER2	Negative limb; represses CLOCK/BMAL1	Per1/2-mutant mice show high bone mass via loss of leptin–sympathetic inhibition	Mouse	[10]
CRY1/CRY2	Negative limb, light-independent	Modulates osteoclast rhythm	Mouse	[8]
REV-ERBα/β; RORα/β/γ	Accessory stabilizing loop at RORE elements	Regulates Bmal1 transcription and stabilizes the accessory clock loop; direct skeletal consequences of pharmacological modulation remain uncertain	Mouse; cell lines	[5]
Melatonin (AANAT/ASMT)	Pineal indoleamine and signal of biological night	Supports osteoblast-mediated bone formation in genetic models; pineal Tph1 deletion reduces bone formation	Mouse	[11]
MT1 (MTNR1A)	Gαi-coupled GPCR	Mtnr1a-null mice have normal bone mass; contributes to ER-stress resistance in cell lines	Mouse; hFOB1.19	[11,12]
MT2 (MTNR1B)	Gαi/Gαq-coupled GPCR	Required for normal bone accrual; low bone mass in Mtnr1b-null mice; null osteoblasts unresponsive to melatonin	Mouse	[11]
MEK1/2, MEK5	MAPK kinase cascades	Required for melatonin-induced osteoblastogenesis in human MSC cocultures	Human MSC/PBMC	[13]
SIRT1/SIRT3	NAD^+^-dependent deacetylases	Mediate melatonin protection from oxidative stress; SIRT3 required in OVX rat model	Rat; MC3T3-E1; human BMSC	[14,15,16]
Sclerostin (SOST)	Osteocyte-derived Wnt antagonist	Circadian Sost regulation in rodents; human serum rhythm not demonstrated	Mouse; human	[17,18]
RANKL/OPG (TNFSF11/TNFRSF11B)	Osteoblast/osteocyte cytokine pair	Rhythmic in mice under BMAL1 control; ratio determines osteoclastogenesis	Mouse; human coculture	[8,13,19]

Note: Gene symbols are italicized, in upper case for human and with an initial capital for mouse and rat, following HGNC and MGI; protein names are set in roman in both species; melatonin receptor proteins are written MT1 and MT2 with a subscript numeral and their genes are cited by symbol. AANAT, arylalkylamine N-acetyltransferase; ASMT, acetylserotonin O-methyltransferase; GPCR, G-protein-coupled receptor; OVX, ovariectomized; PBMC, peripheral blood mononuclear cell.

**Table 2:** Melatonin signals by bone cell type, separated by receptor dependence, with concentration and evidence tier.

Cell Type/System	Receptor Dependence	Basis for Attribution	Downstream Pathway	Functional Effect	Melatonin Concentration	Ref.
Human MSC/PBMC coculture	Receptor-dependent (MT2)	4-P-PDOT, PD98059, BIX02189 all block effect	MEK1/2, MEK5, ERK1/2, ERK5; RUNX2 ↑, PPARγ ↓	Osteoblastogenesis↑; osteoclastogenesis↓ (layered only); OPG:RANKL ↑	50 nM (pharmacological/receptor-active)	[13]
Rodent/human BMSC	Receptor-dependent (MT2)	4-P-PDOT antagonism abolishes effect	Wnt/β-catenin ↑; GSK-3β ↓	Osteogenesis ↑; adipogenesis ↓	100 µM (high pharmacological)	[20]
Rodent/human BMSC	Receptor-dependent, unreplicated	Single study only	lncRNA H19/miR-541-3p/adiponectin	Reinforces β-catenin	100 µM (high pharmacological)	[20]
MC3T3-E1 preosteoblast	Unresolved—no receptor control	Noggin and PD98059 define pathway, not receptor	BMP/ERK/Wnt5a/β-catenin; GSK-3β ↓	Differentiation and mineralization ↑	12.5–100 nM; key experiments at 50 nM (pharmacological/receptor-active)	[21]
Preosteoblast (BMP-4-induced)	Receptor dependence unresolved	H89 and Gö6976 establish PKA/PKC dependence, not receptor identity	Ubiquitin–proteasome inhibition → Osterix (SP7) stabilization	Late-stage mineralization ↑	1 µM (pharmacological)	[22]
Osteoblast/BMSC, oxidative stress	Likely receptor-independent	Antioxidant action; no receptor control	SIRT1/SIRT3 ↑ → SOD2 deacetylation; p66Shc ↓; SOD, GPx ↑	Apoptosis ↓; ALP/mineralization ↑	1–100 µM; rat 50 mg/kg/day	[14,15,16]
hFOB1.19, high glucose	Receptor-implicated	Pharmacological receptor evidence; single cell-line study	PERK–eIF2α–ATF4–CHOP ↓	ER-stress apoptosis ↓	100 nM (pharmacological)	[19]
RAW264.7 osteoclast precursor	Receptor-independent	MT1/MT2 lost on differentiation; supra-saturating concentration	ROS ↓; NF-κB ↓; MAPK/JNK ↓; NFATc1 ↓; ATP6V0D2/DC-STAMP ↓	Osteoclastogenesis and resorption ↓	100 µM (~10^5^× physiological)	[23,24]
BMSC → osteoclast (paracrine)	Receptor-dependent (MT2)	MT2-mediated NF-κB inactivation	RANKL ↓, OPG ↑	OPG/RANKL ratio ↑; osteoclastogenesis ↓	10–100 nM (pharmacological; receptor-active)	[12]
Whole bone/osteoblast lineage	Clock-intrinsic; melatonin arm unresolved	Genetic evidence for BMAL1; pharmacological evidence for melatonin	Reduced osteocyte viability in Bmal1-null bone; SIRT1/SIRT3 protection demonstrated mainly in osteoblast-lineage models	Clock disruption affects bone-cell survival; a direct osteocyte melatonin mechanism is not established	High-µM exposure in pharmacological studies	[14,18,25]

Note: ↑, activation or upregulation; ↓, inhibition or downregulation. Operational exposure categories used throughout this review, defined once in Section 2.4 and applied identically here and in the figure legends, are approximately 0.1–1 nM (physiological, endocrine), >1–10 nM (supraphysiological), >10 nM–1 µM (pharmacological), and >1 µM (high pharmacological). These categories are not formally standardized and should be interpreted together with receptor occupancy, exposure duration, and assay context. ALP, alkaline phosphatase; GPx, glutathione peroxidase; 4-P-PDOT, 4-phenyl-2-propionamidotetralin.

**Table 3:** Randomized controlled trials of melatonin with skeletal or musculoskeletal endpoints, separated by intervention type.

Trial (Year)	Intervention Type	Population	Intervention	*n*	Follow Up	Primary Endpoint	Result	Evidence Quality/Principal Limitation	**Ref.**
MelaOst (2015)	Monotherapy	Postmenopausal osteopenic women, 56–73 yr	Melatonin 1 mg (*n* = 20) or 3 mg (*n* = 20) vs. placebo (*n* = 41), nightly	81	12 mo	BMD by DXA, QCT, HR-pQCT	Small femoral-neck aBMD increase vs. placebo, significant at 3 mg; volumetric spine BMD ↑ at higher dose; turnover markers unchanged	Single small trial; 20 participants per active arm; multiple skeletal sites assessed; independent replication required	[26]
MOPS (2012)	Monotherapy	Perimenopausal women, 45–54 yr	Melatonin 3 mg (*n* = 13) vs. placebo (*n* = 5), nightly	18	6 mo	Calcaneal ultrasound BMD; osteocalcin, NTX; QoL	No significant change in bone density or turnover markers; QoL and menstrual regularity improved	Very small; 4:1 allocation; calcaneal ultrasound not DXA; femoral neck not measured; null for skeletal endpoints	[27]
MOTS (2017)	Combination (4 agents)	Postmenopausal osteopenic women, 49–75 yr	Melatonin 5 mg + strontium citrate 450 mg + vitamin D_3_ 2000 IU + vitamin K_2_ (MK-7) 60 µg (*n* = 11) vs. placebo (*n* = 11)	22	12 mo	BMD lumbar spine and left hip	Lumbar spine +4.3%, femoral neck +2.2%; total hip trend (*p* = 0.069); P1NP ↑, CTX:P1NP ↓	Melatonin contribution not isolable; strontium independently raises DXA aBMD via atomic-number artefact; *n* = 22	[28]
MelaOstrong (2025)	Combination (2 × 2 factorial)	Postmenopausal women with osteopenia	Mock or osteogenic loading × melatonin 5 mg or placebo	19	12 mo	Functional testing, DXA, and exploratory biomarkers	Functional decline occurred only in the mock-loading/placebo group; no clear between-group BMD benefit attributable to melatonin	Small factorial pilot; exploratory analyses; not powered for BMD, bone strength, or fracture	[29]
Thalassaemia RCT (2025)	Monotherapy, secondary osteoporosis	Iron-overloaded thalassaemia, low BMD; mean age 30.8 yr; 80.4% transfusion-dependent	Melatonin 20 mg (*n* = 21) vs. placebo (*n* = 20), nightly	41	12 mo	BMD; turnover markers; MDA; pain	Between-group lumbar-spine BMD not significant (*p* = 0.069); within-group vs. baseline *p* = 0.029; P1NP and MDA ↓ at 6 mo; back pain ↓	Randomized BMD comparison was null; within-group change does not establish efficacy; limited generalizability	[30]

Note: ↑, activation or upregulation; ↓, inhibition or downregulation. No trial was powered for fracture prevention. Combination and factorial designs do not isolate the independent effect of melatonin. aBMD, areal bone mineral density; CRP, C-reactive protein; MDA, malondialdehyde; NTX, N-terminal telopeptide.

**Table 4:** The bone multicellular unit: cell types, circadian evidence, and melatonin evidence.

Cell Type	Role Within the BMU	Circadian Evidence	Melatonin Evidence
Osteoblasts/BMSCs	Matrix synthesis; RANKL and OPG production; determine remodeling balance	Strong—cell-autonomous BMAL1 requirement; the osteoblast clock regulates resorption [25,32]	Strong—MT2 required in Mtnr1b-null osteoblasts; MEK1/2–MEK5 antagonism in primary human cells [11,13]
Osteoclasts	Resorption; determine activation frequency and resorption depth	Strong—Ctsk-Cre Bmal1 deletion impairs osteoclastogenesis [8]	Direct effects mainly at ≥10 µM and probably receptor-independent [24]; indirect MT2-linked OPG/RANKL effect at 10–100 nM [12]
Osteocytes	Mechanosensation; sclerostin and RANKL; initiate targeted remodeling	Rodent Sost rhythmicity; no confirmed human circulating sclerostin rhythm [17]	Provisional—no receptor-defined mechanism demonstrated in primary osteocytes
Bone lining cells	Form the BMU canopy; digest residual matrix; regulate access of precursors [66]	Not separately assessed—existing Cre drivers do not distinguish lining cells from osteoblasts	None identified—evidence gap
Osteomacs	Canopy component; support osteoblast mineralization and coupling [66]	Macrophages have robust autonomous clocks; BMAL1 regulates macrophage IL-1β via NRF2	Melatonin has established anti-inflammatory macrophage effects, but no osteomac-specific skeletal data—evidence gap
Vascular endothelium	Type H (CD31hiEMCNhi) capillaries couple angiogenesis to osteogenesis; supply the BMU [67]	Endothelium is strongly rhythmic in other vascular beds; skeletal endothelium not characterized	No skeletal type H data identified—evidence gap
Marrow adipocytes	Compete with osteoblasts for a common progenitor; secrete adipokines	Adipogenic–osteogenic balance is clock-sensitive in aged BMSCs [58]	RUNX2 increased and PPARγ decreased in primary human cells; fate-switch effect [13]
Marrow immune cells	Activated T and B lymphocytes are a RANKL source; modulate resorption	Immune function is broadly rhythmic	Immunomodulatory effects well described outside bone; skeletal contribution untested

Note: entries marked as evidence gaps indicate that no dedicated circadian or melatonin study in that cell type was identified, not that an effect has been excluded. BMSC, bone marrow mesenchymal stromal cell; BMU, bone multicellular unit; CD31hiEMCNhi, high co-expression of CD31 and endomucin.

**Table 5:** Epidemiological evidence linking circadian disruption to bone loss and fracture.

Study	Design	Population	Exposure	Outcome	Effect Estimate	Principal Limitation	Ref.
Nurses’ Health Study (2009)	Prospective cohort	Female nurses	≥20 yr rotating night shifts	Wrist and hip fracture (8 yr follow-up)	RR 1.37 (95% CI 1.04–1.80); BMI < 24 and never-HRT: RR 2.36 (1.33–4.20)	Association not apparent at 12 yr after single baseline assessment; self-reported exposure; residual confounding acknowledged	[76]
UK Biobank (2025)	Prospective cohort	276,774 participants; 5906 osteoporosis events	Current night-shift work	Incident osteoporosis	HR 1.29 (95% CI 1.12–1.50) for usual night shifts; trend *p* < 0.001	Healthy-worker effect; volunteer cohort; exposure misclassification	[77]
UK Biobank (2025), secondary outcome	Same cohort	As above	As above	Osteoporosis-related pathological fracture	HR 1.88 (95% CI 1.20–3.94)	Wide CI and imprecise secondary outcome; coded pathological fracture is not synonymous with fragility fracture	[77]
UK Biobank (2025), lifetime schedules	Same cohort	75,120 with occupational history	>10 yr duration; 3–8 shifts/month	Osteoporosis	OR 1.21 (0.92–1.58), not significant; OR 1.38 (1.11–1.72)	Non-monotonic across exposure categories	[77]
Review syntheses (2019, 2022)	Narrative/systematic	Adults, mixed	Short sleep, night-shift work, obstructive sleep apnea	BMD, turnover, fracture	Overall direction suggests adverse skeletal associations, but estimates and outcomes are heterogeneous	Heterogeneous exposure definitions; no pooled quantitative estimate	[72,78]

Note: Causal inference remains limited by heterogeneous exposure definitions, residual confounding, and sparse repeated assessment. Absence of interaction with polygenic risk indicates no detected effect modification by genotype; it does not establish robustness to confounding. The competing and concurrent pathways, including the non-causal ones, are displayed in Figure 8.

**Table 6:** Attainable exposure and safety considerations for high-dose melatonin in a skeletal indication.

Domain	Evidence	Implication for a Skeletal Indication
Doses in the literature reviewed here	Randomized trials used 1–20 mg nightly (Table 3); rodent studies commonly used 10–50 mg/kg/day (Section 8.1); in vitro antiosteoclastic effects were observed at 10 µM–1 mM (Section 2.4, Table 2).	The three tiers of the literature are separated by orders of magnitude, and the highest tier has no human counterpart.
Attainable plasma exposure	Physiological nocturnal concentrations are approximately 0.26–0.65 nM (Section 2.4). Reported Cmax after 100 mg orally is of the order of 0.4 µM (≈101,000 pg/mL) and decays within a few hours; absolute oral bioavailability is approximately 9–33%, with large interindividual variation in the absorbed fraction [64].	Sustained exposure at or above 1 µM is not achievable by conventional oral dosing. The 100 µM exposures in Section 3.1, Section 4.1 and Section 4.2 exceed the peak of a 100 mg dose by more than two orders of magnitude.
Allometric gap in the preclinical data	The 50 mg/kg/day rat regimen in the SIRT3 experiment [16] corresponds to a body-surface-area-adjusted human equivalent of roughly 8 mg/kg, or about 550–600 mg daily for a 70 kg adult.	This is approximately 30-fold the highest dose used in any melatonin bone trial (20 mg) and is outside the range in which melatonin has been characterized in humans.
Tolerability at ≥10 mg	A systematic review and meta-analysis of randomized trials of melatonin ≥10 mg found an increased rate of adverse events such as drowsiness, headache and dizziness (rate ratio 1.40, 95% CI 1.15–1.69) without a detectable excess of serious adverse events (0.88, 0.52–1.50) or of withdrawals; however, only four trials met prespecified low risk-of-bias criteria and 37% of studies did not report adverse events at all [79].	Short-term tolerability is reassuring, but the evidence is too sparse to support chronic administration over the multi-year horizon on which osteoporosis is treated.
Glucose homeostasis	MT2 is expressed on pancreatic β-cells, and the MTNR1B variant rs10830963 is a robust glycemic and type 2 diabetes locus [71] (Section 10). Exogenous melatonin impairs glucose tolerance in a genotype-dependent manner, the effect being confined to carriers of the risk allele in a randomized crossover study [80].	The variant proposed in Table 7 as a candidate stratification marker is also a marker of susceptibility to a metabolic adverse effect. Evening dosing coincides with any late carbohydrate intake, and dysglycemia is prevalent in the target population.
Sedation, sleep and falls	Daytime sleepiness and next-morning residual sedation are the most consistently reported adverse effects [79]; Section 7.5 and Section 12 note that melatonin improves sleep and that no trial was designed to partition direct skeletal effects from changes in sleep, activity, or fall risk.	The endpoint of interest is fracture, and most fragility fractures follow a fall. At high chronic doses the sleep pathway becomes a potential liability as well as a potential confounder.
Duration and special populations	No skeletal trial exceeded 12 months (Table 3), and long-term safety in children and adolescents is unresolved. Melatonin is a CYP1A2 substrate, with clearance altered by fluvoxamine, caffeine and smoking [64].	Extrapolation to chronic use, to younger or growing skeletons, and to polypharmacy in older adults is not supported. Any future high-dose trial should specify a pharmaceutical-grade product.

Note: Cmax, maximum plasma concentration; CYP1A2, cytochrome P450 1A2. Human-equivalent doses are body-surface-area adjusted. This table addresses the exposures implied by the micromolar mechanisms reviewed here; it is not a general safety assessment of conventional low-dose melatonin.

**Table 7:** Candidate stratification variables for a chronobiological research framework, with current validation status and research priority.

Domain	Marker/Instrument	Proposed Research Use	Validated Against a Skeletal Outcome?
Chronotype	Munich ChronoType Questionnaire; Morningness–Eveningness Questionnaire	Stratify participants by circadian phase preference in trial design	No direct validation of the MCTQ or MEQ for skeletal treatment stratification; a recent single-center observational study reported an association between circadian-rhythm disruption and osteoporosis [81,82]
Endogenous melatonin	24 h urinary 6-sulfatoxymelatonin; salivary dim-light melatonin onset	Identify low-output individuals as a candidate enriched population	Partial—urinary aMT6s rhythmicity has been related to urinary NTx rhythmicity in a small cohort of blind women; DLMO and aMT6s remain unvalidated for BMD, fracture, or treatment-response enrichment [83,84]
Cortisol rhythm	Diurnal serum or salivary cortisol	Identify HPA-driven bone loss as a competing mechanism	Established for bone loss caused by endogenous or exogenous glucocorticoid excess; not validated as a predictor of skeletal response to melatonin [69,70]
Bone-turnover rhythm	Nocturnal and morning β-CTX, P1NP	Characterize remodeling rhythmicity and candidate pharmacodynamic responses	Partial—β-CTX rhythmicity is established, including under constant-routine conditions; P1NP rhythmicity is weak or absent, and predictive value for treatment response remains untested [53,54]
Genetic	MTNR1B rs10830963; exploratory clock-gene variants	Exploratory pharmacogenetic hypothesis only	No skeletal pharmacogenetic validation—rs10830963 is an established glycemic/type 2 diabetes variant, whereas clock-gene variants remain exploring bone outcomes [34,71]
Wearables	Actigraphy; sleep tracking	Objective phenotyping of chronodisruption at scale	Partial observational validation—actigraphy rest–activity and sleep measures have been associated with BMD or bone-turnover markers in some cohorts, with null findings in others; no treatment-response threshold is validated [73,74,75]
Drug timing	Administration time relative to endogenous rhythm	Optimize efficacy of existing agents	Yes for a small teriparatide timing trial [79]; no skeletal timing evidence for melatonin or bisphosphonates [85,86,87]

References identify representative supporting publications and do not imply that the listed marker is clinically validated for patient stratification. aMT6s, 6-sulfatoxymelatonin; DLMO, dim-light melatonin onset; MEQ, Morningness–Eveningness Questionnaire; MCTQ, Munich ChronoType Questionnaire. This table defines a validation agenda; with the single exception noted for teriparatide timing, none of these variables is currently validated for clinical stratification. A research-priority rating (high, intermediate, or exploratory) aligned with the ranking in Section 11.1 is as follows: endogenous melatonin and bone-turnover rhythm, high; drug timing, high for teriparatide and untested for melatonin; cortisol rhythm and wearables, intermediate; chronotype and genetic variants, exploratory. The MTNR1B allele is a safety as well as an efficacy variable, since it also marks susceptibility to melatonin-induced impairment of glucose tolerance (Section 8.4).

## Data Availability

No new data were created or analyzed in this study. Data sharing is not applicable to this article. The full search strings supporting Section Evidence Identification and Appraisal are provided.

## Supplementary Materials

The following supporting information can be downloaded at: https://www.mdpi.com/article/10.3390/biom16091232/s1, Table S1. The four operational exposure categories used throughout this review are summarized.

## Abbreviations

Nomenclature convention: gene symbols are italicized, in upper case for human (*BMAL1*, *MTNR1B*) and with an initial capital for mouse and rat (*Bmal1*, *Mtnr1b*), following HGNC and MGI; protein names are set in roman in both species; melatonin receptor proteins are written as MT1 and MT2 with a subscript numeral, and their genes are cited by symbol. *BMAL1* is the HGNC-approved symbol; *ARNTL* is the former symbol.

AANAT	arylalkylamine N-acetyltransferase
AFMK	N^1^-acetyl-N^2^-formyl-5-methoxykynuramine
ALAN	artificial light at night
ALP	alkaline phosphatase
ALPL	alkaline phosphatase gene
AMK	N^1^-acetyl-5-methoxykynuramine
ARE	antioxidant response element
ASMT	acetylserotonin O-methyltransferase
ATF4	activating transcription factor 4
BMAL1 (ARNTL)	brain and muscle ARNT-like 1
BMD	bone mineral density
BMP	bone morphogenetic protein
BMSC	bone marrow mesenchymal stromal cell
BMU	bone multicellular unit
Cmax	maximum plasma concentration
CHOP	C/EBP-homologous protein
CK1δ/ε	casein kinase 1δ/ε
CRY	cryptochrome
CTX (β-CTX)	C-terminal telopeptide of type I collagen
DXA	dual-energy X-ray absorptiometry
eIF2α	eukaryotic initiation factor 2α
ER	endoplasmic reticulum
ERK	extracellular signal-regulated kinase
FGF-23	fibroblast growth factor 23
FOXO	Forkhead box O
GBD	Global Burden of Disease
GSK-3β	glycogen synthase kinase-3β
HPA	hypothalamic–pituitary–adrenal
HED	human-equivalent dose
HO-1	heme oxygenase-1
KEAP1	Kelch-like ECH-associated protein 1
HR-pQCT	high-resolution peripheral quantitative computed tomography
ipRGC	intrinsically photosensitive retinal ganglion cell
JNK	c-Jun N-terminal kinase
LRP5/6	low-density lipoprotein receptor-related protein 5/6
MAPK	mitogen-activated protein kinase
MEK	mitogen-activated protein kinase kinase
MSC	mesenchymal stromal cell
MT1 (MTNR1A)/MT2 (MTNR1B)	melatonin receptor 1/2
NFATc1	nuclear factor of activated T cells c1
NF-κB	nuclear factor kappa B
NTX	N-terminal telopeptide
NOX1	NADPH oxidase 1
NQO1	NAD(P)H quinone dehydrogenase 1
NRF2	nuclear factor erythroid 2-related factor 2
OPG	osteoprotegerin
P1NP	procollagen type I N-terminal propeptide
PBMC	peripheral blood mononuclear cell
PERK	protein kinase R-like ER kinase
PKA/PKC	protein kinase A/C
PPARγ	peroxisome proliferator-activated receptor γ
QCT	quantitative computed tomography
RANK(L)	receptor activator of NF-κB (ligand)
ROR	retinoic acid receptor-related orphan receptor
RORE	ROR response element
ROS	reactive oxygen species
RUNX2	runt-related transcription factor 2
SCN	suprachiasmatic nucleus
SIRT	sirtuin
SOD	superoxide dismutase
SOST	sclerostin gene
SP7	Osterix
TCF/LEF	T-cell factor/lymphoid enhancer factor
TPH1	tryptophan hydroxylase 1
TRAF6	TNF receptor-associated factor 6
YAP	Yes-associated protein
