# Supplementary material for: The Circadian–Melatonin Axis in Bone Remodeling: Receptor-Dependent Signaling, Receptor-Independent Actions, and Translational Constraints
Source: Biomolecules. 2026 Aug 25;16(9):1232. doi: 10.3390/biom16091232 (PMC13604420; doi:10.3390/biom16091232)
Supplement: Supplementary file 1 [file biomolecules-16-01232-s001.zip › biomolecules-4491552-supplementary.pdf]

Table S1. The four operational exposure categories used throughout this review are summarized.

Operational category	Concentration	Interpretation
Physiological (endocrine)	~0.1–1 nM	Within or close to the nocturnal plasma range of healthy adults; receptor-mediated signalling expected.
Supraphysiological	>1–10 nM	Above the endogenous plasma range but well within high-affinity MT_1/MT_2 binding; receptor-mediated signalling remains the most plausible mechanism.
Pharmacological	>10 nM – 1 μM	Attainable only by exogenous administration; receptor-mediated and receptor-independent actions may both contribute.
High pharmacological	>1 μM	Four to six orders of magnitude above plasma; receptor-independent antioxidant and intracellular chemistry increasingly likely to dominate.
